# DiCoExpress: a tool to process multifactorial RNAseq experiments from quality controls to co-expression analysis through differential analysis based on contrasts inside GLM models

**DOI:** 10.1186/s13007-020-00611-7

**Published:** 2020-05-12

**Authors:** Ilana Lambert, Christine Paysant-Le Roux, Stefano Colella, Marie-Laure Martin-Magniette

**Affiliations:** 1 LSTM, Laboratoire des Symbioses Tropicales et Méditerranéennes, IRD, CIRAD, INRAE, SupAgro, Univ Montpellier, Montpellier, France; 2 Institute of Plant Sciences Paris-Saclay (IPS2), Université Paris-Saclay, CNRS, INRAE, Univ Evry, Bat. 630, 91405 Orsay, France; 3 Institute of Plant Sciences Paris Saclay (IPS2), Université de Paris, CNRS, INRAE, Bat. 630, 91405 Orsay, France; 4 UMR MIA-Paris, AgroParisTech, INRAE, Université Paris-Saclay, 75005 Paris, France

**Keywords:** RNA-seq, Analysis workspace, Differential expression, Contrasts, Co-expression

## Abstract

**Background:**

RNAseq is nowadays the method of choice for transcriptome analysis. In the last decades, a high number of statistical methods, and associated bioinformatics tools, for RNAseq analysis were developed. More recently, statistical studies realised neutral comparison studies using benchmark datasets, shedding light on the most appropriate approaches for RNAseq data analysis.

**Results:**

DiCoExpress is a script-based tool implemented in R that includes methods chosen based on their performance in neutral comparisons studies. DiCoExpress uses pre-existing R packages including FactoMineR, edgeR and coseq, to perform quality control, differential, and co-expression analysis of RNAseq data. Users can perform the full analysis, providing a mapped read expression data file and a file containing the information on the experimental design. Following the quality control step, the user can move on to the differential expression analysis performed using generalized linear models thanks to the automated contrast writing function. A co-expression analysis is implemented using the coseq package. Lists of differentially expressed genes and identified co-expression clusters are automatically analyzed for enrichment of annotations provided by the user. We used DiCoExpress to analyze a publicly available RNAseq dataset on the transcriptional response of *Brassica napus L.* to silicon treatment in plant roots and mature leaves. This dataset, including two biological factors and three replicates for each condition, allowed us to demonstrate in a tutorial all the features of DiCoExpress.

**Conclusions:**

DiCoExpress is an R script-based tool allowing users to perform a full RNAseq analysis from quality controls to co-expression analysis through differential analysis based on contrasts inside generalized linear models. DiCoExpress focuses on the statistical modelling of gene expression according to the experimental design and facilitates the data analysis leading the biological interpretation of the results.

## Background

During the last decades, Next-Generation Sequencing (NGS) technologies have developed at a fast pace with the improvement of data quality coupled with a reduction of experimental costs. Since the early years of NGS, the use of RNAseq to profile transcriptomes became the method of choice replacing in time microarray-based analyses [1]. Plant biologists use RNAseq-based transcriptomic extensively, generating knowledge about transcriptional regulation in several biological processes [2–5]. Differential gene expression analysis across different experimental conditions is classically used to gain insight into gene regulation events and gene co-expression analysis to identify functional modules.

A classical analysis workflow starts with a data normalization step to account for technical biases that affect the number of reads mapped to a gene. Several methods are available, and among the most used, we can find RPKM (Reads Per Kilobase per Million mapped reads) [6], Upper quartile normalization [7], RLE (Relative Log Expression) [8] and TMM (Trimmed Mean of the M-values) [9]. Multiple methods, based on different statistical modelling of data, are available to perform differential expression analysis. Negative binomial-based models with robust mean–variance modelling, have been used extensively at the beginning, and they are available in the R-packages edgeR [10] and DESeq [11]. More recently, the linear models and their generalized extensions for negative binomial distributions (GLM) have been proposed to account for the versatility of multifactorial experiments. They are available in the R-package limma [12] for the linear models and in the R-packages edgeR [10] and DESeq2 [11] for the generalized linear models. Following differential gene expression analysis, several approaches to identify and group co-expressed genes have been in use over the years. Pearson’s or Spearman’s correlations, WGCNA (Weighted correlation network analysis) method [13], hierarchical clustering and K-means are the most conventional approaches found in the literature [14, 15]. With these approaches, the number of clusters is chosen, either a priori or a posteriori, by the user. Mixture models offer a different approach by identifying an underlying structure which corresponds to clusters of co-expressed genes. Moreover, a model selection criterion allows determining the most appropriate cluster number [16, 17].

To perform such analysis, tools associated with the methods are available in R to quite quickly get from data to results [18]. In parallel bioinformatics tools offering a Graphical User Interface (GUI), and interactive visualization tools were developed. First-generation tools included the RNAseq read-mapping step [19–21], more often realized independently at present, depending on data and genome availability. Several of these GUI tools [22–28] ease the use of the main RNAseq analysis R-packages for normalisation and differential expression analysis such as limma [12], DESeq [9], DESeq2 [11] and edgeR [10]. The role of these GUI is to realize R-based RNAseq data analysis with little or no experience in the command line. More recent tools take advantage of the R-shiny framework that eases the creation of a GUI for R-packages and pipelines [29]. The majority of these GUI tools includes a high number of data visualisation options and the possibility to generate figures for publications.

Even with all these tools, a biologist is often facing a dilemma on how to analyse his dataset correctly. Indeed a characteristic shared by the majority of GUI tools developed up to date is to offer the user the possibility to choose among multiple statistical methods for each step of the analysis with no specific propositions. However, the RNAseq data specificities, such as heterogeneity of counts or overdispersion among biological replicates, represent a methodological challenge that has to be addressed by proper statistical modelling of the gene expression. It is worth noting that in the case of multifactorial experiments, if interaction terms are included in the modelling, the writing of the contrasts might become tricky, requiring a good understanding of some statistical concepts, not always mastered by a biologist. As a result, the large-scale data analysis of RNAseq data is not straightforward for a biologist.

To respond to some of these challenges in RNAseq data analysis, we developed DiCoExpress to analyse RNAseq projects with at most two biological factors and an unbalanced number of replicates for any condition.

### Implementation

DiCoExpress is a script-based tool implemented in R with a set of directories where data, scripts and results are organised. The potential users of DicoExpress should know what a linear model and a contrast to be tested in such a context are. Regarding programming skills, the users should know how to use a function in R, identifying required and optional arguments.

### Selection of the statistical methods

DiCoExpress offers a validated set of methods, based on three neutral comparison studies [17, 30, 31]. The idea of such studies is to design and implement a framework to generate realistic benchmark datasets with known truth to make an objective and reproducible performance assessment. Comparing normalisation methods, Dillies et al. [30] showed that the RLE method implemented in the package DESeq2 [11] and the TMM method implemented in the package edgeR [10] demonstrate satisfactory behaviour in the presence of highly expressed genes. Both these methods maintain a reasonable false-positive rate without loss of power. The choice of both methods was confirmed by Reddy et al. [32] and Evans et al. [33] even in experiments with slightly asymmetric differential expression or different amounts of mRNA/cell per condition. Based on these detailed evaluations, both RLE and TMM are suitable, but we decided to choose the TMM normalisation as the default method and proposed RLE as an alternative due to the choice made for the differential analysis described below.

Rigaill et al. [31] made a neutral comparison study among differential gene expression methods, including negative binomial-based, generalized linear models, and linear models on transformed data. Performance analyses based on the p-value distributions, ROC curves, and proportion of true and false-positive rates show a clear difference of behaviour between negative binomial-based methods and the others. Linear models on transformed data or generalized linear models are consequently the most adapted for the differential analysis. Among these models, as also observed in Schurch et al. [34], when the proportion of differentially expressed genes is low, the results obtained with the method implemented in the edgeR package are more satisfying. We thus chose the statistical model implemented in the edgeR package as a method of choice for differential expression data analysis. Moreover, we propose automatic writing of a large number of contrasts in order to facilitate the comparisons between the biological conditions considered in the experimental design. This automatic writing is a real advantage because, in the available R-packages, most contrasts in GLM with interactions between two factors must be handwritten and require thus an excellent understanding of the statistical modelling.

For the co-expression analysis, we preferred mixture models to correlation-based approaches. Mixture models aim at identifying an underlying structure in modelling the unknown distribution by a weighted sum of parametric distributions, each one representing a group of co-expressed genes. Gaussian mixture models were relevant for microarray data and were applied with success on several datasets [35, 36]. For RNAseq data, which are discrete, Rau et al. [16] first concluded that normalized expression profiles modelled with a Poisson mixture are relevant for co-expression analysis. However, in the Poisson mixture, the dependence structure between samples is not considered and can mislead the results. To tackle this problem, they proposed then a Gaussian mixture after a transformation of the normalized expression profiles [17]. This model seems to be more suitable for RNAseq co-expression analysis by providing a proper identification of the groups of co-expressed genes because it accounts for per-cluster correlation structures among samples. For these reasons, we chose this Gaussian mixture implemented in the coseq R-package [17].

### Architecture of DiCoExpress

**Fig. 1:**
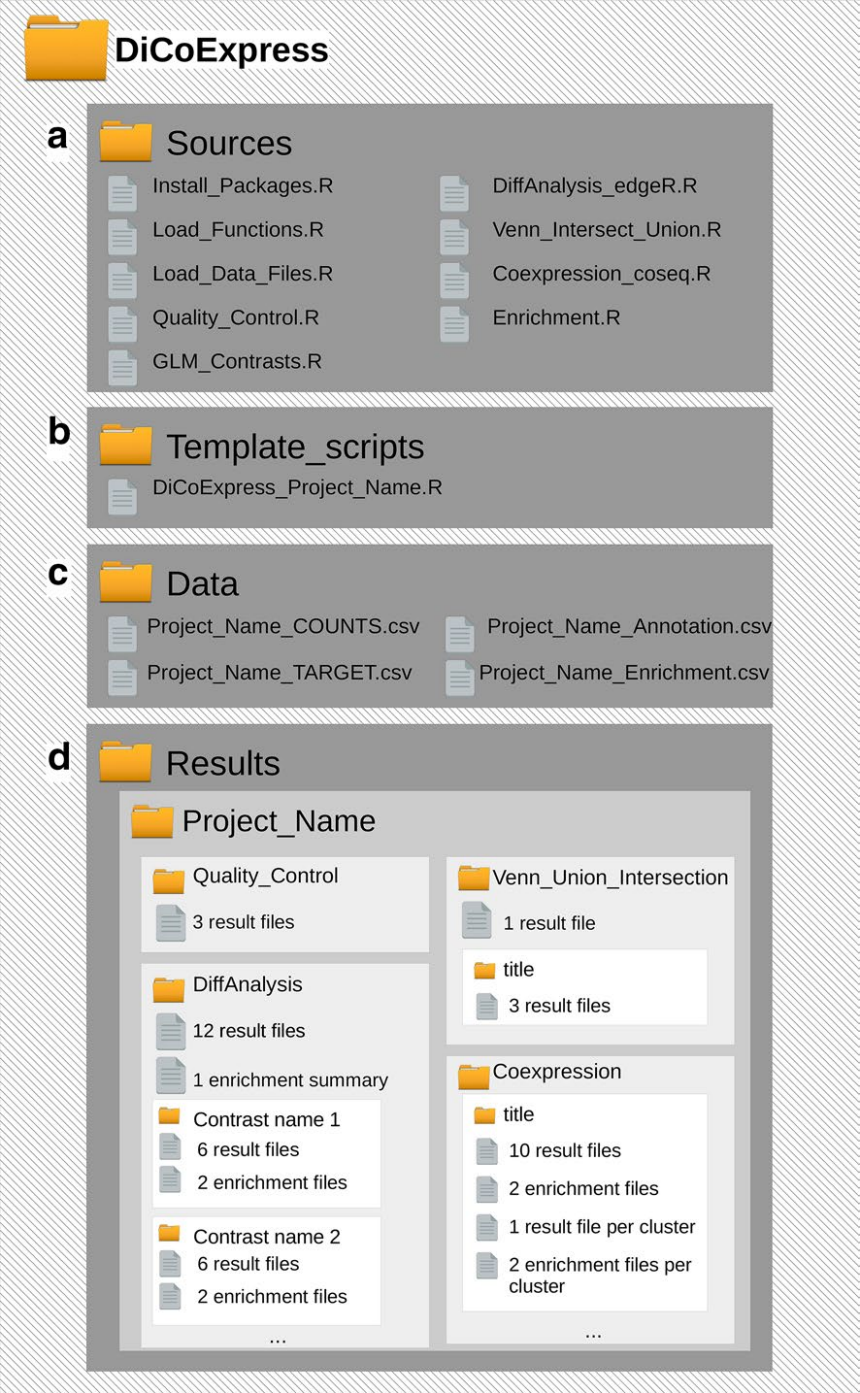
DiCoExpress workspace. A visual representation of the DiCoExpress Workspace. **a** Sources directory contains all R functions implemented in DiCoExpress. **b** Template_scripts directory is the directory where an analysis script for each project analyzed must be saved. **c** Data directory is the directory where for each project, the input files (target and count tables) must be saved. If an annotation file used to describe biologically the different result tables as well as an reference file for the enrichment analysis are available, they must be also be saved in this directory. **d** Results directory contains all results obtained for each project

Using these neutral comparison studies, we combined the most suitable methods for each step of a standard RNAseq analysis. The tool DiCoExpress is a directory composed of a set of subdirectories that is to be installed on a computer for analysing RNAseq datasets (see Fig. 1). The directory *Data* stores all the projects, and the directory *Results* contains a subdirectory per project with all the results of the different steps. The directory *Sources* contains the R functions used by DiCoExpress. Finally, the directory *Template_scripts* contains an R script file for each project, allowing a semi-automated data analysis where the user is guided through all the steps from normalisation to co-expression analysis. With DiCoExpress, our objective is to automate the statistical analysis of gene expression according to the experimental design to ease the interpretation of the results in biological terms.

**Fig. 2:**
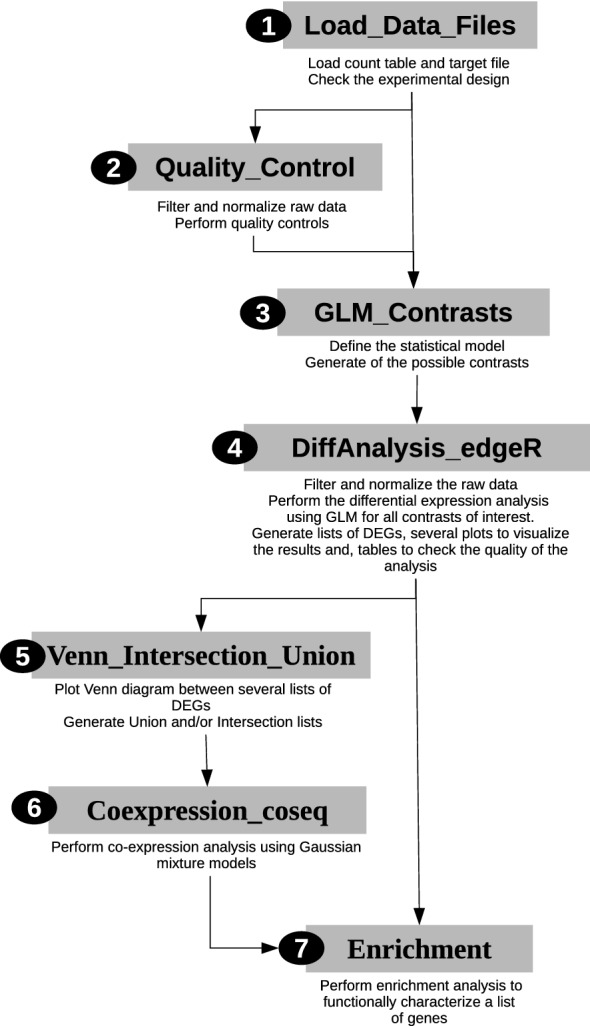
Overview of DiCoExpress workflow. DiCoExpress is composed of seven functions written in R programming language and available in the directory Sources. After loading the input files with the (1) Load_Data_Files function, a data quality control is done with the (2) Quality_Control function. Differential expression analysis using generalized linear models is performed by using the (3) GLM_Contrasts and (4) DiffAnalysis_edgeR functions. The union or intersection lists of differentially expressed genes are generated by the (5) Venn_Intersection_Union function. A co-expression analysis is performed on these lists by the (6) Coexpression_coseq function. Finally, the functional characterisation of lists of genes is tested by using the (7) Enrichment function

To create DiCoExpress, we use the R programming language and several R-packages from CRAN and Bioconductor [37, 38]. Each step of the analysis has a dedicated function available in the directory *Sources*. Seven functions compose the core of DiCoExpress (Fig. 2), and they are combined in a script, stored in the directory *Template_scripts* for each project to specify the steps of the analysis and the parameters to use. A full description of these seven functions is available in the Reference Manual (Additional file 1).

### Input files and data quality controls

To use DiCoExpress on a project, the user has to provide only two input files: one containing a count table summarizing the mapped reads for each gene, named Project_Name_COUNTS.csv, and a second one with a description of the project design according to the experimental factors, named Project_Name_TARGET.csv. If functional gene annotations are available in a file, the user has the option to upload it. This information is integrated into the result tables and can also be used to perform enrichment tests.

The (1) Load_Data_Files function allows the user to upload the Project_Name_COUNTS.csv and Project_Name_TARGET.csv files. A check is done to be sure that both files are adequately built: the samples in Project_Name_COUNTS.csv file must be organized in the same order as the rows of the Project_Name_TARGET.csv. If it is inconsistent, then the columns of the Project_Name_COUNTS.csv are reorganized according to the column of the target file. DiCoExpress performs analysis for a complete experimental design. If this condition is not verified, then an error message appears, and the script stops running. A filter option in Load_Data_Files is proposed to extract or remove a subset of samples, thus avoiding manual modifications of the expression file. The filtering rules are described according to the Project_Name_TARGET.csv (see section Results for an example).

The (2) Quality_Control function produces a PDF file containing graphical outputs before and after normalisation: histograms of the library sizes, boxplots of the counts for each sample, heatmap, and principal component analysis (PCA), as shown in Additional file 2. This step is optional, but we advise the users to perform it to evaluate the quality of the RNAseq data before further analyses.

### Differential expression analysis

**Fig. 3:**
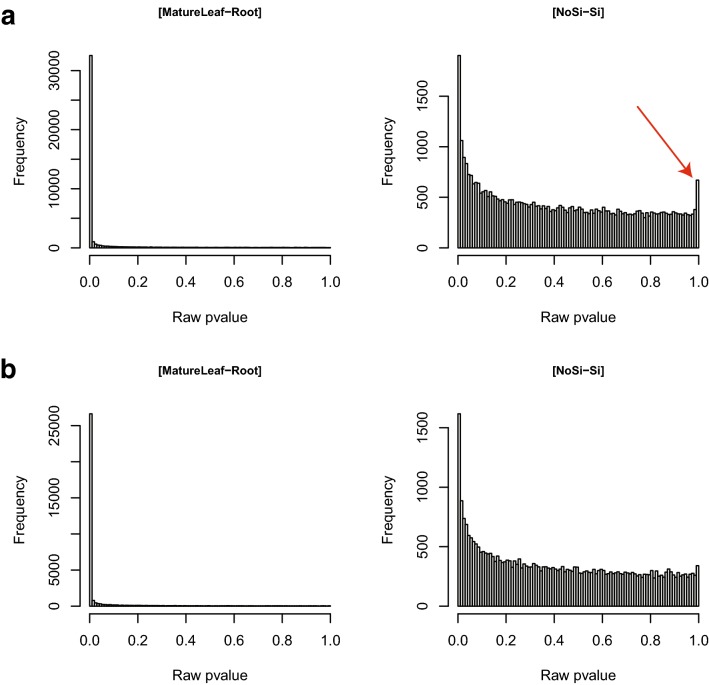
Histograms of raw p-values *Brassica napus* analysis. Histograms of the raw p-value for the contrasts [MatureLeaf–Root] and [NoSi–Si] according to the CPM_Cutoff parameter when the filtering strategy is NbConditions **a** CPM_Cutoff = 1 (default argument). **b** CPM_Cutoff = 5

The differential analysis is based on a negative binomial GLM, where the log of the gene expression is modelled by all the factors describing the experiment. When the number of observations is two times greater than the number of parameters of the model, we advise to include interaction terms between the biological factors. Such terms in the gene expression definition might reveal meaningful interactions such as genotype-environment interaction and answer in a direct way to some biological questions [39–41]. The (3) GLM_Contrasts function automatically writes a list of contrasts based on the model specified by the user. We focused on contrasts involving the biological factors, and their names are sufficiently explicit to understand the associated biological question addressed. For example, we proposed automatic writing of the difference between two modalities of a biological factor averaged on the second factor or for a given modality of the second factor. Running this function is a prerequisite to run the differential expression analysis. The (4) DiffAnalysis_edgeR function uses edgeR R-package to estimate the parameters of the GLM and then test every contrast chosen by the user. As proposed by Rigaill et al. [31], the distribution of raw p-values of each contrast is inspected to assess the quality of the statistical modelling of the gene expression. Since the distribution of raw p-values is theoretically dominated by a uniform distribution, the fit between the statistical model and the data can be observed on these raw p-value histograms. If the raw p-value distribution is not satisfactory (see example in the section Results and Fig. 3), we advise repeating the analysis using a more stringent cut-off for the filtering step or another rule of filtering. If the raw p-value distribution remains unsatisfactory, the problem might come from a large number of parameters compared to the number of observations available to estimate them. In this case, we advise modifying the modeling of the gene expression removing, for example, the interaction term. For each contrast, a subdirectory is created to store information about all the tested genes and also about the differentially expressed genes (DEGs). The function also generates graphical outputs about the behaviour of the top list of the DEGs (Additional file 3). All these files are described in details in the Reference Manual (Additional file 1).

### Co-expression analysis

The (5) Venn_Intersection_Union function helps the user in the interpretation of the results by comparing different DEG lists. This function also generates the union and/or the intersections of these DEG lists to perform a co-expression analysis with the (6) Coexpression_coseq function. This latter function uses coseq R-package [17] to transform the raw data into normalised expression profiles. We kept the filter function of coseq removing the genes with low mean normalised counts. Those discarded genes are assigned in Cluster 0. A co-expression analysis is performed on the remaining genes using a Gaussian mixture after an arcsin transformation of the normalised expression profiles. Practically, multidimensional Gaussian mixtures of 5–30 subpopulations with unequal proportions and general covariance matrix are estimated. The EM algorithm used to estimate the model parameters is known to be sensitive to the initialisation point. Coseq uses a small-EM strategy, and in DiCoExpress, we go further to get robust results. First, mixture models with 5, 10, 15, 20, 25, and 30 subpopulations are estimated 5 times each to identify an interval for the final number of co-expressed gene clusters. A second collection of models on this interval of a subpopulation is then estimated 40 times each (per default). The best mixture model is the one minimising the Integrated Completed Likelihood (ICL). The ICL curve is expected to be a convex function of the number of subpopulations, and we use this criterion to assess that the chosen model fits well the data. When a different behaviour of the ICL curve is observed, we advise the user to modify the dataset removing some genes that show a too flat normalised profile. For the co-expression analyses, we recommend using a powerful calculation server. The RData object of the second loop is saved for each mixture model so, if the function is stopped, the analysis can be resumed. The RData of the selected model is also saved. Moreover, several tables and graphics are saved to check the analysis quality and to explore the co-expression results. An example is discussed in the section Results, and all the files are described in details in the Reference Manual (Additional file 1).

### Enrichment analysis

Once an RNAseq statistical data analysis is complete, researchers often evaluate the coherence of the results by comparing them with biological knowledge. To this end, the (7) Enrichment function performs hypergeometric tests to find annotation terms that are specifically over or under-represented in a given list of genes with respect to a reference specified by an annotation file. The enrichment analysis can be performed both on the lists of differentially expressed genes, and the co-expressed gene clusters.

## Results and discussion

We illustrate the use of DiCoExpress by analysing a dataset associated with the publication of Haddad et al. [42]. This RNAseq dataset describes gene expression in roots and mature leaves of Brassica napus with or without silicon (Si) treatment. Three biological replicates are available. The experimental design can be described by two biological factors Tissue and Treatment, and a technical factor Replicate with three modalities (Table 1). To illustrate the outputs of enrichment tests, we used the GO annotation of B. napus v.5 from the Brassica genome database [43, 44] to perform enrichment analyses.

**Table 1:** Target table of *Brassica napus* dataset in R

Sample	Tissue	Treatment	Replicate
MatureLeaf_NoSi_R1	MatureLeaf	NoSi	R1
MatureLeaf_NoSi_R2	MatureLeaf	NoSi	R2
MatureLeaf_NoSi_R3	MatureLeaf	NoSi	R3
MatureLeaf_Si_R1	MatureLeaf	Si	R1
MatureLeaf_Si_R2	MatureLeaf	Si	R2
MatureLeaf_Si_R3	MatureLeaf	Si	R3
Root_NoSi_R1	Root	NoSi	R1
Root_NoSi_R2	Root	NoSi	R2
Root_NoSi_R3	Root	NoSi	R3
Root_Si_R1	Root	Si	R1
Root_Si_R2	Root	Si	R2
Root_Si_R3	Root	Si	R3

The target table provides the experimental design. Each row describes a sample by specifying the level for each factor in the columns

We tested DiCoExpress on the full dataset available in contrast to Haddad et al, who only focused on the root samples. The procedure is described in Additional file 4 as a tutorial of DiCoExpress. We started the analysis by filtering not expressed genes and those with low counts. We used the Counts Per Million (CPM) method with CPM_Cutoff = 1 and Filter_Strategy = “NbConditions” that are the default arguments of Quality_Control function. We choose the default method TMM to normalise the RNAseq libraries. Checking the quality control results in Brassica_napus_Data_Quality_Control.pdf output file, we observe a higher number of reads in the mature leaf samples compared to the root samples; nonetheless, the normalisation seems suitable for further analysis since the boxplots of normalised counts are almost similar across all the samples (Additional file 2: Fig. A, B). A hierarchical clustering heatmap and principal component analysis graphs are generated to look at the sample similarities. In our analysis, we observe, as expected, a clear difference between the two tissues as well as an apparent clustering of mature leaf samples according to the treatment (Additional file 2: Fig. C, D).

We performed a differential expression analysis using a GLM with both biological factors and the technical replicate factor. We included an interaction between the two biological factors in the model. We checked the quality by looking at the raw p-value histograms of the seven contrasts automatically written by the GLM_Contrasts function. For the three contrasts, [MatureLeaf-Root], [NoSi_MatureLeaf-NoSi_Root], and [Si_MatureLeaf-Si_Root], the end of the histograms of raw p-values corresponds to a uniform distribution indicating a good fit of the GLM model. However, on the histograms of the four other contrasts, [NoSi-Si], [MatureLeaf_NoSi-MatureLeaf_Si], [Root_NoSi-Root_Si] and [MatureLeaf_NoSi-MatureLeaf_Si]-[Root_NoSi-Root_Si], we observe an increase of the frequency around 1: this usually suggests that data are not properly filtered (Fig. 3a). Following this observation, we went back to the beginning of the analysis, setting a more stringent CPM_Cutoff = 5, and we obtained satisfying raw p-value histograms for the seven contrasts (Fig. 3b). We observed, as expected that the highest number of differentially expressed genes is found for the comparison of both tissues with 28 261, 25 734, and 25 757 DEGs for the contrasts [MatureLeaf-Root], [NoSi_MatureLeaf-NoSi_Root] and [Si_MatureLeaf-Si_Root], respectively. A small number of differentially expressed genes is identified between the two treatments: 218, 754 and 173 DEGs for the contrasts [NoSi-Si], [MatureLeaf_NoSi-MatureLeaf_Si] and [Root_NoSi-Root_Si], respectively (Additional file 3: Fig. A). An advantage of using a GLM with an interaction term is to identify straightforward genes that respond differently to the Silicon treatment in the two tissues using the [MatureLeaf_NoSi-MatureLeaf_Si]-[Root_NoSi-Root_Si] contrast. In this interaction analysis, we found 106 genes differentially expressed, and an example of a gene in this list is shown in Additional file 3: Fig. B. The hierarchical clustering on the top 50 DEGs ranking on their p-values for this contrast is also proposed by DiCoExpress (Additional file 3: Fig. C). On the bottom of this plot, we observe groups of genes with a clear opposite behaviour between the two tissues. For the others, the behaviour is more variable, but all these genes are declared to be the most impacted genes by the treatment and in different ways in the two tissues.

**Fig. 4:**
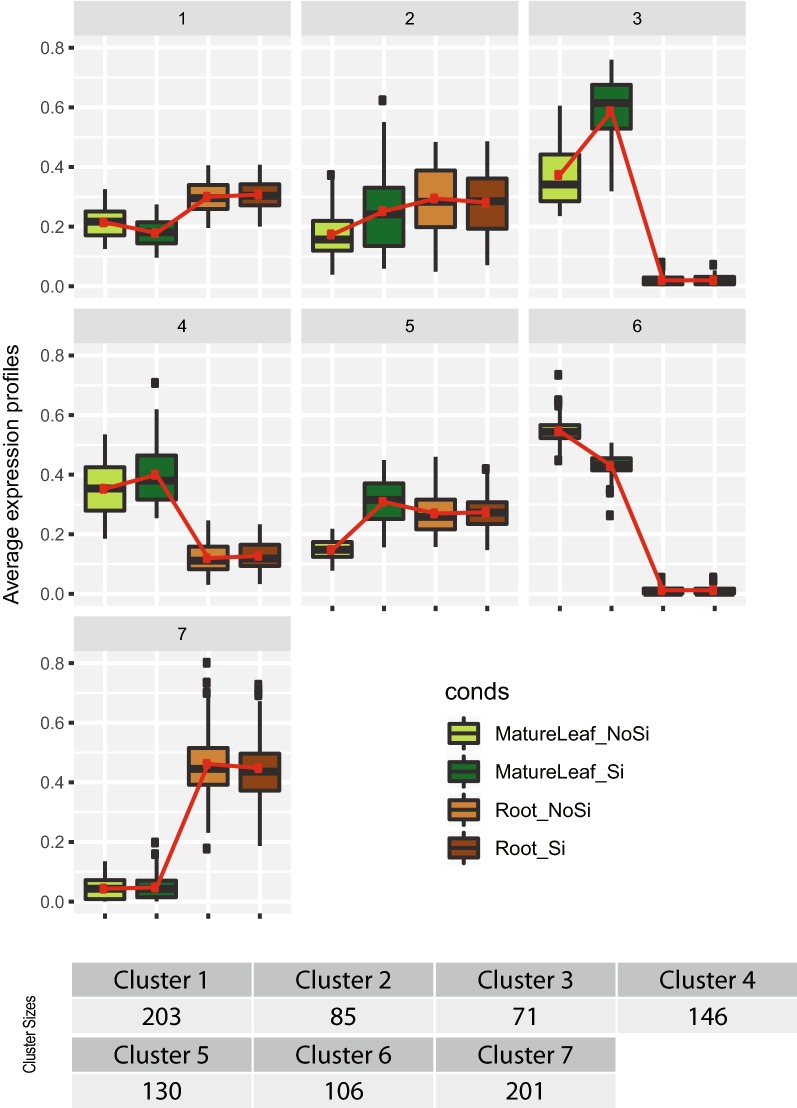
Co-expression clusters for the *Brassica napus* analysis. Average expression profiles and size of the c co-expression clusters found by analysing of the 945 Differentially expressed genes (DEGs) from the union of three contrasts: [MatureLeaf_NoSi–MatureLeaf_Si], [Root_NoSi–Root_Si] and [MatureLeaf_NoSi–MatureLeaf_Si]-[Root_NoSi–Root_Si]

As users often need to compare DEG lists, in DiCoExpress, we propose the Venn_Intersection_Union function to generate these lists quickly. In the *Brassica napus* dataset, we unite three contrasts: [MatureLeaf_NoSi-MatureLeaf_Si], [Root_NoSi-Root_Si] and [MatureLeaf_NoSi-MatureLeaf_Si]-[Root_NoSi-Root_Si] to study genes impacted in their transcription by the treatment (Additional file 5: Fig. A). Within the Venn diagram, we can distinguish genes whose expression varies in response to treatment in a specific tissue or in both treatments with a similar or different behaviour depending on the tissue and examples from each class is shown in Additional file 5: Fig. B. This grouping of genes using a Venn diagram is based only on the results of the single contrast differential analysis. However, by performing a co-expression analysis, we can go further in the interpretation by clustering these genes according to their average expression profile in all samples. We applied the Coexpression_coseq function with default parameters to group the 945 DEGs from the union of the three contrasts. The ICL curve has a clear minimum which is a marker of a good quality clustering analysis (Additional file 4), and seven clusters of co-expressed genes are found (Fig. 4). Three genes with low mean normalised counts were assigned in Cluster 0, i.e., coseq could not assign them to a cluster. Clusters 3 and 6 (71 and 106 genes, respectively) contain genes with low expression and no change in the roots, but their expression varies in response to the Si treatment in the mature leaves (over-expression in Cluster 3 and under-expression in Cluster 6). Conversely, Cluster 7 (201 genes) show low expression with no significant change in the mature leaves, but they are strongly expressed in the roots with a slight reduction following the treatment. Cluster 1 and 4 (203 and 146 genes, respectively) include genes more expressed in one tissue compared to the other one (higher expression in mature leaves for cluster 4 and higher expression in roots for cluster 1) but without significant Si treatment response. In cluster 2 (85 genes) and cluster 5 (130 genes) are grouped genes showing a small difference in expression levels between the two tissues. For both clusters, genes show over-expression following the treatment in leaves (more apparent in cluster 5), but no significant change in roots. The cluster composition based on probabilistic modelling of the normalised gene profiles is, as it could be expected, different from the groups of genes found with the DEG list comparisons (Additional file 6). Following the co-expression analysis, that finishes the statistical analysis of this dataset used to illustrate the use of DiCoExpress, we performed enrichment analyses on these 7 clusters, and also of the 106 DEGs for the interaction contrast, and they are available in the tutorial (Additional file 4). Further interpretation and discussion of the biology behind these enrichments are beyond the scope of our presentation of the DiCoExpress usage.

## Conclusions

DiCoExpress is an R script-based tool for analysing efficiently multifactorial RNAseq transcriptome experiments from quality controls to co-expression analysis through differential expression analysis. We based the development of DiCoExpress on neutral comparison studies combining the most performant statistical approaches for each step of a standard RNAseq analysis. The choice of a statistics defined framework, limiting the free selection of models and methods, was deliberate to propose to the users the possibility to perform RNAseq analysis in a standardised manner. In DiCoExpress, we used generalized linear models implemented in the R-package edgeR for differential gene expression analysis and Gaussian mixture models implemented in the R-package coseq to perform the co-expression analysis. DiCoExpress simplifies the GLM analysis proposing automated writing of all possible contrasts and optimises the co-expression analysis with the re-estimation of the collection of Gaussian models. DiCoExpress produces a collection of files to visualise the results and multiple summary files of the data for further data exploration. The integrated enrichment analysis with the hypergeometric test gives the user the first glimpse at potential biological functions underlying the different gene lists. In conclusion, DiCoExpress allows the user to perform RNAseq data analysis with validated statistical methods with a set of R-scripts in a pre-existing and organised working environment.

## Availability and requirements

- Project name: DiCoExpress
- Project home page: https://forgemia.inra.fr/GNet/dicoexpress
- Operating system(s): Windows, Mac OS, Linux
- Programming language: R
- Other requirements: R version 3.5.0 or higher with FactoMineR_2.0
- License: GPL-2 | GPL-3
- Any restrictions to use by non-academics: None

## Supplementary information

**Additional file 1.** Reference Manual.

**Additional file 2.** Data quality control *Brassica napus* results. Data quality control after the filtering and TMM normalisation (A) Library sizes for each sample (B) Boxplot of normalised counts for each sample (C) Heatmap made with the Euclidean distance and the Ward distance to cluster the samples (D) First and second axes of the Principal Component Analysis on the normalised counts. Samples corresponding to mature leaves are coloured in green and those of roots in brown.

**Additional file 3.** Differential expression *Brassica napus* analysis results. Differential expression analysis results with (A) the barplot of Up and Down differentially expressed genes for each contrast, (B) an example of one gene differentially expressed in the interaction contrast. (C) Hierarchical clustering of the top 50 DEGs for the interaction contrast.

**Additional file 4.** Analysis Tutorial.

**Additional file 5.***Brassica napus* GLM contrasts results. Organisation of the genes impacted in their transcription by the silicon treatment issued from the GLM differential analysis. (A) Venn diagram describing the numbers of differentially expressed genes in the three contrasts [MatureLeaf_NoSi-MatureLeaf_Si], [Root_NoSi-Root_Si] and [MatureLeaf_NoSi-MatureLeaf_Si]-[Root_NoSi-Root_Si], and their overlaps: (B) Gene expression of genes representative of each group of the Venn diagram (described with small letters from a to g).

**Additional file 6.** Comparison of DEG lists and clusters of co-expression.

## Data Availability

https://forgemia.inra.fr/GNet/dicoexpress.

## Abbreviations

CPM: Counts per million
DEGs: Differentially expressed genes
FDR: False discovery rate
GLM: Generalized linear models
ICL: Integrated completed likelihood
LM: Linear model
NB: Negative binomial
NGS: Next generation sequencing
PCA: Principal component analysis
RLE: Relative log expression
RPKM: Reads per kilobase per million mapped reads
TMM: Trimmed mean of the M-values
