# Supplementary material for: DiCoExpress: a tool to process multifactorial RNAseq experiments from quality controls to co-expression analysis through differential analysis based on contrasts inside GLM models
Source: Plant Methods. 2020 May 12;16:68. doi: 10.1186/s13007-020-00611-7 (PMC7216733; doi:10.1186/s13007-020-00611-7)
Supplement: Supplementary file 1 — Additional file 1. Reference Manual. [file 13007_2020_611_MOESM1_ESM.pdf]

DiCoExpress
REFERENCE MANUAL

April 2020

Ilana Lambert, Christine Paysant-Le Roux, Stefano Colella and Marie-Laure Martin-Magniette

What is DiCoExpress?

DiCoExpress is a workspace developed in R for scientists, not experts in statistics, and with knowledge of R, for the analysis of RNA-seq datasets. DiCoExpress performs quality controls, proposes generalized linear model (GLM) analyses, with automated contrasts writing, allowing the user to answer to several biological questions. Finally, DiCoExpress conducts a coexpression analysis using mixture models. Statistical results of the differential analysis, as well as the coexpression analysis, can be completed with gene annotations. Annotations enrichment against a reference set (the whole genome or a gene subset) can be tested.

How to install DiCoExpress?

After downloading DiCoExpress from https://forgemia.inra.fr/GNet/dicoexpress, either as a zip file or using the command "git clone" in Shell.

1. Open R or Rstudio (if installed) with R version >=3.5.0 in the directory DiCoExpress

2. Install all CRAN R packages used in DiCoExpress by running the R installation program: `source(‶./Sources/Install_Packages.R″)`. If all packages are successfully installed, the logical value “TRUE” is printed on the R Console pane for each package. If the logical value “FALSE” is printed, you need to check the error messages in the Console panel to find a solution.

WARNING: we advise Windows users to install DiCoExpress in the home directory to avoid possible errors when the results are saved. This potential problem is due to the path length limitation in some Windows OS versions.

How to use DiCoExpress?

Input files

Two data files are required:

1) The raw counts table file that contains the expression level for the genes (rows) in each sample (columns). The sample names must begin by a letter and not by a number. The first column heading is *Gene_ID*. This file must be named: **Project_Name_COUNTS.csv**

2) The target table, created by the user, provides the experimental design. Each row of the target table describes a sample by specifying the level for each factor (columns) explaining the experimental conditions. The first column of the target file indicates the sample names with heading *Sample*, and the last column must be named *Replicate*. The biological factors are be placed in-between these two compulsory columns and named by the user in line with the experimental condition. To avoid errors during the script execution, it is preferable that each modality description starts with a letter (for example, for a factor Month, it is better to write M1 rather than 1). This file must be named: **Project_Name_TARGET.csv**

Two optional files can also be provided:

3) A file named **Project_Name_Annotation.csv** with the first column labeled *Gene_ID.* This file contains the gene identifiers used in the count table. The second column, named *Gene_Name,* contains the name / short function description. Each gene is described by only one line. This information is added to the different output files of the DiCoExpress data analysis.

4) A file named **Project_Name_Enrichment.csv** file is required to perform enrichment tests. It contains in the first column the gene identifier named *Gene_ID* and in the second column named *Annotation* the annotation term (GO, KEGG pathway, others) corresponding to a given gene. The second column contains only one annotation term per line. If a gene has multiple annotations, its identifier must be repeated in the first column as many times as its number of annotations. If a gene has no annotation, it must appear in the GO file with an "NA" in the *Annotation* column.

WARNING: the character # is the comment character for the R software and must not be used in these files.

Examples of these 4 input files are presented in the directory *Data* for the analysis of a *Brassica napus* project.

Template_script

For each project of analysis, a script must be created by the user and placed in the directory *Template_scripts*.

This template contains the R command lines and parameters required by the DiCoExpress functions. The file DiCoExpress_Brassica_napus.R in the directory *Template_scripts* is an example of such a script, and it can be copied and modified as needed to generate a template for a new project analysis.

In this script, user runs at first a set of commands:

- to load the functions of DiCoExpress (description of functions below)

```
source("../Sources/Load_Functions.R")
Load_Functions()
```

- to set Working, Data, and Results directories:

```
Working_Directory <- ".."
Data_Directory <- paste0(Working_Directory,"/Data")
Results_Directory <- paste0(Working_Directory,"/Results")
```

- to give a project a name of his choice:

```
Project_Name <- "User_Project_name"
```

Then the script is used to launch the functions of DiCoExpress described in detail hereafter.

What are the functions of DiCoExpress?

1. Load_Data_Files

This function loads the two compulsory files **Project_Name_COUNTS.csv** and **Project_Name_TARGET.csv** and re-organizes the target file so that its rows (*sample IDs*) are organized in the same order of the columns of the raw count table. If they are provided by the user, the function also loads **Project_Name_Annotation.csv** and **Project_Name_Enrichment.csv**.

The function also proposes a filter option to extract a subset of samples and adapts the target file as needed. Finally, this function checks whether the experimental design is complete and balanced, contains up to two biological factors, and whether the last column is named *Replicate*.

This function has 4 arguments:

- **Data_Directory**: the directory containing the input files.
- **Project_Name**: the name of the project, used as a prefix for each result file, is the same one used in the names of **.COUNTS.csv** and **.TARGET.csv** files.
- **Filter**: a list of filter rules and the name of the new project that is generated

using them. A filter rule is described by 2 character strings and a logical value:

`c("Name_of_factor","level_of_this_factor",TRUE or FALSE).`

TRUE means that only the modality of this factor is kept, and FALSE means that this modality is removed. If the first string is `″Sample″` and the second string a name of one sample, then this sample is removed from the expression and target tables. The last element of the list must be a string of characters to give a name to the project on the filtered dataset.

By default, `Filter=NULL`, the whole dataset is analyzed.

- **Sep**: the field separator character of the input files. By default, `Sep=″,″`

To use this function, users run the following command line:

`Load_Data_Files(Data_Directory, Project_Name, Filter, Sep)`

This function returns up to 5 elements: the target table, the count table, the project name, as well as if available the annotation and the enrichment files.

2. Quality_Control

This function filters and normalizes the raw data and checks the quality of the data before and after normalization. Data filtering removes genes with no expression and with low counts. These low expressed genes interfere in the parameter estimation of the generalized linear model (GLM) used during the differential analysis. To remove the low expressed genes, different strategies are proposed using a “counts per million (CPM) cutoff”. The normalization of data using the calcNormFactors function in edgeR

is performed to eliminate biases between library sizes. A PDF file containing graphical outputs before and after normalization: histograms of the library sizes, boxplots of the counts for each sample, heatmap of the euclidean distances with a link of Ward, and principal component analysis (PCA) are proposed to the user to check the quality of the dataset.

This function has 10 arguments:

- **Data_Directory**: the directory containing the input files.
- **Results_Directory**: the directory containing the output files.
- **Project_Name**: the name of the project, used as a prefix for each result file.
- **Target**: the target table (output of Load_Data_Files function).
- **Raw_Counts**: the raw count table (output of Load_Data_Files function).
- ~~**Annotation_FileName**: the name of the annotation file.~~
- **Filter_Strategy**: A string of characters. The possible values are `"NbConditions"`, `"NbReplicates"` or `“filterByExpr”` respectively corresponding to the number of biological conditions, the number of replicates of the dataset, and the function filterByExpr from the edgeR package. The two formers mean that only genes with cpm greater than or equal to the set `CPM_Cutoff` in at least NbConditions or NbReplicates samples are kept. The third option allows the use of the function filterByExpr from the edgeR package with the default parameters.
- **Color_Group**: vector of colors for the graphical outputs. By default, `Color_Group=NULL` and colors are automatically proposed according to the biological conditions.
- **CPM_Cutoff**: the filtering cutoff for the cpm method. By default, `CPM_Cutoff=1`, genes whose cpm expression is greater than `CPM_Cutoff` in x samples (depending on the `Filter_Strategy` used) are kept.
- **Normalization_Method**: The normalization method to be used corresponding to the method argument of the calcNormFactors function of the R-package edgeR. By default, `Normalization_Method = “TMM”`.

To use this function, run the following command line:

```
Quality_Control(Data_Directory, Results_Directory,
Project_Name, Target, Raw_Counts, Filter_Strategy, Color_Group,
CPM_Cutoff, Normalization_Method)
```

The Quality_Control function returns 3 output files saved in the directory *Results/Project_Name/Quality_Control:*

- **Project_Name_Normalization_Results.txt**
  A text file containing the number of samples, the number of genes, and the normalization factors.
- **Project_Name_Low_count_genes.txt**
  A text file containing the list of low expressed genes.
- **Project_Name_Data_Quality_Control.pdf**
  A PDF file containing graphical outputs before and after normalization.

3. GLM_Contrasts

This function defines the generalized linear model (GLM) that is used to analyze the dataset. The statistical model can be written with or without a replicate factor and with or without an interaction factor if two biological factors are available.
This function automatically generates several possible contrasts for the differential expression analysis. Three relevant types of contrasts for RNAseq analyzes in biology are possible:
- The effect of one biological factor averaged on the second biological factor;
- The effect of one biological factor given a level of the second biological factor;
- The interaction effect between the two biological factors.

This function has 5 arguments:

- **Results_Directory**: the directory containing the output files.
- **Project_Name**: the name of the project, used as a prefix for each result file.
- **Target**: the target table (output of Load_Data_Files function)
- **Replicate**: if TRUE, a replicate term is added in the GLM.
- **Interaction**: if TRUE, an interaction between biological factors is added in GLM

To use this function, run the following command line:

`GLM_Contrasts(Data_Directory, Results_Directory, Project_Name, Target, Replicate, Interaction)`

The GLM_Contrasts function returns 3 output files saved in the directory *Results/Project_Name/DiffAnalysis* :

- **Project_Name_GLM_Contrasts.txt**
  A text file containing the numbered list of contrast.
- **Project_Name_GLM_Model.txt**
  The design matrix of the generalized linear model.
- **Project_Name_Contrasts_Matrix.txt**
  A table containing the coefficients for each contrast to be tested equal to zero.

4. DiffAnalysis_edgeR

This function estimates the parameters of the GLM and performs differential analysis for all contrasts. This function is based on functions available in the R-package edgeR. First data are filtered and normalized, then parameters of the GLM are estimated, and a likelihood ratio test (LRT) is performed for each contrast considered. The probabilities of significance (p-values) generated by the LRT are adjusted by the Benjamini-Hochberg procedure (BH).

This function has 15 arguments:

- **Data_Directory**: the directory containing the input files.
- **Results_Directory**: the directory containing the output files.
- **Project_Name**: the name of the project, used as a prefix for each result file.
- **Target**: the target table (output of Load_Data_Files function)
- **Raw_Counts**: the raw count table (output of Load_Data_Files function)
- **GLM_Model**: GLM matrix (output of GLM_Contrasts function)
- **Contrasts**: Contrasts matrix (output of GLM_Contrasts function)
- **Index_Contrast**: The vector of numbers corresponding to the number of contrasts of interest. This number is found in Project_Name_GLM_Contrasts.txt file. By default, all the possible contrasts are analyzed.
- ~~**Annotation_FileName**: The name of the annotation file.~~
- **Filter_Strategy**: A string of characters. The possible values are `"NbConditions"`, `"NbReplicates"` or `“filterByExpr”` respectively

corresponding to the number of biological conditions, the number of replicates of the dataset, and the function filterByExpr from the edgeR package. The two formers mean that only genes with cpm greater than or equal to the set `CPM_Cutoff` in at least NbConditions or NbReplicates samples are kept. The third option allows the use of the function filterByExpr from the edgeR package with the default parameters.

- **Alpha_DiffAnalysis**: the cutoff used on FDR values to decide if a gene is differentially expressed or not. By default, `Alpha_DiffAnalysis=0.05`.
- **NbGenes_Profiles**: the number of top DEGs for the single gene expression profile. By default `NbGenes_Profiles=20`.
- **NbGenes_Clustering**: the number of top DEGs for the hierarchical clustering. By default `NbGenes_Clustering=50`.
- **CPM_Cutoff**: the filtering cutoff for the cpm method. By default, `CPM_Cutoff=1`, genes whose cpm expression is greater than `CPM_Cutoff` in x samples (depends on the Filter_Strategy used) are kept.
- **Normalization_Method**: The normalization method to be used corresponding to the method argument of the calcNormFactors function of the R-package edgeR. By default, `Normalization_Method = "TMM"`.

To use this function, run the following command line:

```
DiffAnalysis.edgeR(Data_Directory, Results_Directory,
Project_Name, Target, Raw_Counts, GLM_model, Contrasts,
Index_Contrast, Filter_Strategy, Alpha_DiffAnalysis,
NbGenes_Profiles, NbGenes_Clustering, CPM_Cutoff,
Normalization_Method)
```

The DiffAnalysis_edgeR function returns 9 output files saved in the directory *Results/Project_Name/DiffAnalysis:*

- **Project_Name_Contrasts_Interest_Matrix.txt**
  A table containing the coefficients for each contrast of interest.
- **Project_Name_Estimated_Dispersion.txt**
  The tagwise dispersion value for each gene.
- **Project_Name_Fitted_Values.txt**
  The fitted values of each gene in each sample.

- **Project_Name_Raw_pvalue_histograms.pdf**

  Histograms of raw p-values for each contrast analyzed.
- **Project_Name_DiffAnalysis_Comparisons.txt**

  The number of Up and Down expressed genes for each analyzed contrast.
- **Project_Name_Down_Up_DEG.pdf**

  Histogram of Up and Down expressed genes for each analyzed contrast.
- **Project_Name_Compare_table.txt**

  A table summarizing the results of the contrasts of interest. The genes are those differentially expressed at least one time. The value 1 indicates that the gene is DE.
- **Project_Name_NormCounts_log2.txt**

  The normalized and log2 transformed data table for all samples.
- **Project_Name_NormCounts_log2_Mean_SD.txt**

  The mean and standard deviation of normalized and log2 transformed data for each biological condition.

For each contrast analyzed, a subdirectory named as the contrast is created in *Results/Project_Name/DiffAnalysis*. It contains 6 result files specific to the given contrast:

- **Project_Name_ Contrast_LRT_BH.txt**

  For all normalized genes: LogFoldChange (logFC), logCountsperMillion (logCPM), Likelihood ratio (LR), p-value et False Discovery Rate (FDR).
- **Project_Name_ Contrast_DEG_BH.txt**

  For all DEGs: logFC, logCPM, LR, p-values and FDR.
- **Project_Name_ Contrast_Id_DEG.txt**

  For all DEGs: List of gene identifiers (Gene ID).
- ~~**Project_Name_Contrast_DEG_NormCounts.txt**~~

  ~~For all DEGs: Table of normalized counts.~~
- ~~**Project_Name_Contrast_DEG_log2_NormCounts.txt**~~

  ~~For all DEGs: Table of normalized and log2 transformed counts.~~
- **Project_Name_Contrast_plotSmear.pdf**

  Plot the log of the ratio of expression levels for each gene between two experimental groups (the log fold-change) against the overall average expression level for each gene across the two groups (the log-concentration). The DEGs are plotted in red.

- **Project_Name_Contrast_TopXX_Profile.pdf**
  Single gene profile for the top XX among the DEGs. XX is specified by the argument `NbGenes_Profiles`.
- **Project_Name_Contrast_TopYY_Clustering.pdf**
  Hierarchical clustering for top YY DEGs. YY is specified by the argument `NbGenes_Clustering`.

5. Venn_Intersection_Union

This function plot Venn diagram between several lists of DEGs using the vennDiagram function of the limma package. The union or intersection list is generated. Several Venn analyses can be performed on the same project: each analysis is defined from the argument `Groups` and stored in a subdirectory based on the `Title` given to the analysis.

This function has 8 arguments:

- **Data_Directory**: the folder containing the input files.
- **Results_Directory**: the folder containing the output files.
- **Project_Name**: the name of the project, used as a prefix for each result file.
- **Title**: a string of characters used for the prefix of results files and to create a new subdirectory in Results/Project_Name/Venn_Intersection_Union where results are stored. ~~Several Venn analysis can be performed on the same project.~~
- ~~**Annotation_FileName**: the name of the annotation file.~~
- **Groups**: the name of the contrasts used for the Venn analysis. These names must be the same as column names of Compare_table (output of DiffAnalysis_edgeR). The Venn diagram plot is generated for the comparison for a maximum of 5 groups.
- **Operation**: The possible operations are “Union” or “Intersection”. You need to run the function two times if you want to perform both operations.

To use this function, run the following command line:

```
Venn_Intersection_Union(Data_Directory, Results_Directory,
Project_Name, Title, Groups, Operation)
```

The Venn_Intersection_Union function returns 3 output files saved in the directory *Results/Project_Name/Venn_Intersection_Union/Title :*

- **Project_Name_Title_Union_List.txt** or **Project_Name_Title_Intersection_List.txt**
  The gene identifiers (Gene_ID) of the Union or Intersection list.
- **Project_Name_Title_Summary_Table.txt**
  Only for Union analysis, the table containing the identifiers of all genes analyzed. The last column, *"DE_Group",* corresponds to the legend of VennDiagram.
- **Project_Name_Title_Venn_Diagram.pdf**
  The Venn diagram and the legend for the DE groups.

The Venn_Intersection_Union function also returns in *Results/Project_Name/Venn_Intersection_Union*

- **Project_Name_Title_Compare_table.txt**
  This table is one output of the function DiffAnalysis_edgeR, updated with a new Union or Intersection column.

6. Coexpression_coseq

This function performs a coexpression analysis on a list of genes defined with the Venn_Intersection_Union function. ~~differentially expressed genes in at least one contrast~~. This analysis is based on Gaussian mixture models implemented in coseq R-package. Following the recommendations in the package, we use the filter function of coseq to remove the genes with low mean normalized counts. The remaining genes are analyzed after an "arcsin" transformation of the normalized expression profiles. Mixture models from 5 to 30 subpopulations by step of 5 are estimated in a first loop to define a second, more accurate collection of models estimated a large number of times in a second loop. The best model is the one that minimizes the Integrated Completed Likelihood (ICL). Several tables and graphics are proposed to check the quality of the analysis and to explore the results. The RData object of the second loop is saved at each iteration to allow resuming the analysis if it is stopped. The final results are also saved in an RData object to allow exploring them within R.

This function has 10 arguments:

- **Data_Directory**: the directory containing the input files.
- **Results_Directory**: the directory containing the output files.
- **Project_Name**: the name of the project, used as a prefix for each result file.
- **Title**: a string of characters used for the prefix of result files and to create a new subdirectory in Results/Project_Name/Coexpression where results are stored.
- **Target**: the target table (output of Load_Data_Files function)
- **Raw_Counts**: the raw count table (output of Load_Data_Files function)
- ~~**Annotation_FileName**: the name of the annotation file.~~
- **Color_Group**: vector of colors for the graphical outputs. By default, `Color_Group=NULL` and colors are automatically proposed according to the biological conditions.
- **A**: number of iteration for the first loop. By default `A=5`
- **B**: number of iteration for the second loop. By default, `B=40`
- **K**: the collection of models visited in the first loop. By default, `K={5, 10, 15, 20, 25, 30}` meaning that mixtures with 5, 10, … 30 subpopulations are estimated.

To use this function, run the following command line:

```
Coexpression_coseq(Data_Directory, Results_Directory,
Project_Name, Title, Target, Raw_Counts, Color_Group, A, B, K)
```

The Coexpression_coseq function returns 11 output files saved in the directory *Results/Project_Name/Coexpression/Title :*

- **Project_Name_Title_Results_First_Loop.txt**
  The table of loglikelihood and ICL values of the first loop
- **Project_Name_Title_Loop_1.pdf**
  LogLikelihood and ICL curves of the first loop
- **Project_Name_Title_Results_Second_Loop.txt**
  The list of K chosen for the second loop and table of ICL values.
- **Project_Name_Title_Loop_2.pdf**
  The ICL curve of the second loop.
- **Project_Name_Title_coseq_loop_2.Rdata**
  The RData of the second loop. If the analysis is stopped, the user can resume it with this RData.

- **Project_Name_Title_coseq_final.Rdata**

  The RData of the final results. The user can open it in R to explore the results.
- **Project_Name_Title_Results_Final.txt**

  Description of the final results: number of selected clusters and corresponding ICL value, cluster sizes, number of genes with a maximal conditional probability greater than 0.9, and number of genes not included in the coexpression analysis.
- **Project_Name_Title_ClusterN_GeneID.txt**

  List of gene identifiers of cluster N (One file per cluster)
- **Project_Name_Title_AllCusters.txt**

  For each analyzed gene: the annotation, the number of the cluster where it is assigned, and the maximal conditional probability. Genes in Cluster 0 correspond to genes not included in the coexpression analysis (low mean normalized counts).
- **Project_Name_Title_Final_Coseq.pdf**

  A pdf file containing several plots: normalized expression profiles for each sample and cluster, boxplots of normalized expression profiles for each sample and cluster, boxplot, and histograms of maximal conditional probabilities.
- **Project_Name_Title_Boxplot_profiles_Coseq.pdf**

  Boxplot of normalized gene expression profiles for each biological condition and each cluster.

7. Enrichment

This function performs enrichment analysis using the hypergeometric test to characterize a list of genes functionally. This function determines the annotation terms underrepresented, and those overrepresented in the gene list when compared to a reference list (loaded by the function Load_Data_Files, if Enrichment file provided by the user). This analysis can be automatically performed on all coexpression clusters or all the lists of DEGs resulting from the differential analysis according to the value of the `Title` argument.

This function has 6 arguments:

- **Data_Directory**: the folder containing the input files.

- **Results_Directory**: the folder containing the output files.
- **Project_Name**: the name of the project, used as a prefix for each result file.
- **Title**: NULL or a string of characters. ~~used for the prefix of results files. Several enrichment analysis can be performed on the same project.~~ If NULL, the enrichment tests are performed on all the contrasts studied during the differential analysis. If it is a string of characters representing a `Title`, then enrichment tests are performed on all the clusters of coexpression available in the subdirectory Results/Project_Name/Coexpression/Title
- ~~**Contrast_Name**: Contrast_Name=NULL indicates that enrichment analysis is performed on co-expression clusters. Otherwise Contrast_Name is a string of character corresponding to a contrast name (available in the column names of the file Project_Name_Compare_table.txt).~~
- **Reference_Enrichment**: the reference for the enrichment test loaded with the function Load_Data_Files from the Data_Directory. ~~The user can use the genome or the transcriptome as reference file.~~
- **Alpha_Enrichment**: the threshold for the hypergeometric test. This cutoff is used on raw p-values to decide if a given annotation term is over or underrepresented in the gene list compared to the reference. By default, `Alpha_Enrichment=0,01`.

To use this function, run the following command line:

`Enrichment(Data_Directory, Results_Directory, Project_Name, Title, Reference_FileName, Alpha_Enrichment)`

If `Title=NULL`, the Enrichment function returns results in

Results/Project_Name/DiffAnalysis

- **Project_Name_Summary_Enrichment.txt**

  A summary table of the enrichment analysis performed on all the contrasts. If a term is enriched (over- or under-represented), its value = 1, else its value = 0. The last column of the table contains the number of enriched DEG lists.

and 2 output files in the subdirectories of Results/Project_Name/DiffAnalysis

- **Project_Name_[contrast]_All_Enrichment_Results.txt**

  Result table for each term: the number of annotated and not annotated genes in the reference file (Urn_Success and Urn_Failures), the number of annotated

genes in the gene list file (Trial_Success), the number of genes in the gene list (Trial_effective), the percentage of annotated genes in the reference file (Urn_percentage_Success) and the gene list (Trial_percentage_Success) and finally, the raw p-values of hypergeometric test for the depletion and enrichment analysis (Pvalue_over and Pvalue_under respectively).

- **Project_Name_[contrast]_Significant_Enrichments.txt**

  Result table with only terms declared significant (raw p-value < Alpha_Enrichment)

If Title is a string of characters, the Enrichment function returns results in *Results/Project_Name/Coexpression*

- **Project_Name_Title_Summary_Enrichment.txt**

  A summary table of the enrichment analysis performed on all the clusters of coexpression. If a term is enriched (over- or under-represented), its value = 1, else its value = 0. The last column of the table contains the number of enriched clusters.
- **Project_Name_Title_AllClusters_All_Enrichment_Results.txt**

  Result table for each term calculated on all the genes used in the coexpression analysis
- **Project_Name_Title_AllClusters_Significant_Enrichments.txt**

  Result table with only terms declared significant (raw p-value < Alpha_Enrichment)
- **Project_Name_Title_Cluster_X_All_Enrichment_Results.txt**

  Result table for each term calculated on all the genes of the Cluster X
- **Project_Name_Title_Cluster_X_Significant_Enrichments.txt**

  Result table with only terms declared significant (raw p-value < Alpha_Enrichment)
