# Supplementary material for: DiCoExpress: a tool to process multifactorial RNAseq experiments from quality controls to co-expression analysis through differential analysis based on contrasts inside GLM models
Source: Plant Methods. 2020 May 12;16:68. doi: 10.1186/s13007-020-00611-7 (PMC7216733; doi:10.1186/s13007-020-00611-7)
Supplement: Supplementary file 2 — Additional file 2. Data quality control Brassica napus results. Data quality control after the filtering and TMM normalisation (A) Library sizes for each sample (B) Boxplot of normalised counts for each sample (C) Heatmap made with the Euclidean distance and the Ward distance to cluster the samples (D) First and second axes of the Principal Component Analysis on the normalised counts. Samples corresponding to mature leaves are coloured in green and those of roots in brown. [file 13007_2020_611_MOESM2_ESM.pdf]

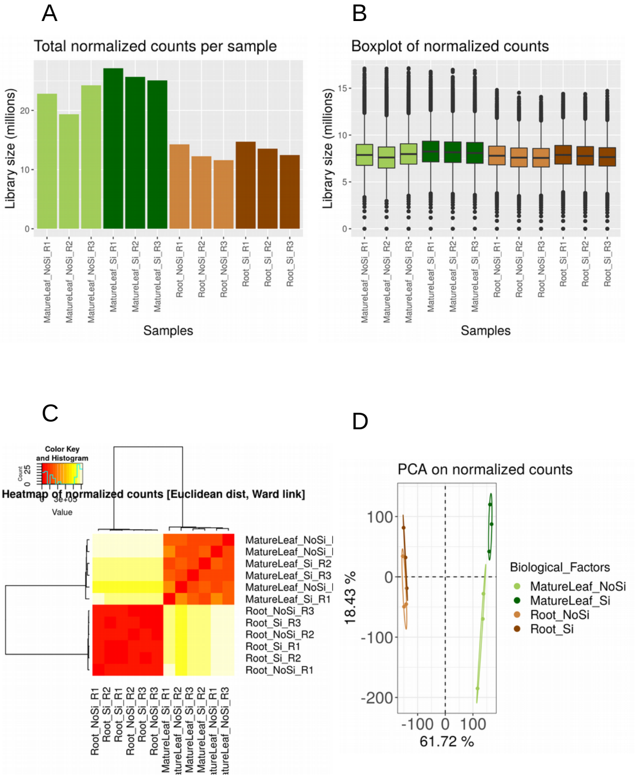
A
Total normalized counts per sample
Library size (millions)
0
10
20
MatureLeaf_NoSi_R1
MatureLeaf_NoSi_R2
MatureLeaf_NoSi_R3
MatureLeaf_Si_R1
MatureLeaf_Si_R2
MatureLeaf_Si_R3
Root_NoSi_R1
Root_NoSi_R2
Root_NoSi_R3
Root_Si_R1
Root_Si_R2
Root_Si_R3
Samples
B
Boxplot of normalized counts
Library size (millions)
0
5
10
15
MatureLeaf_NoSi_R1
MatureLeaf_NoSi_R2
MatureLeaf_NoSi_R3
MatureLeaf_Si_R1
MatureLeaf_Si_R2
MatureLeaf_Si_R3
Root_NoSi_R1
Root_NoSi_R2
Root_NoSi_R3
Root_Si_R1
Root_Si_R2
Root_Si_R3
Samples
C
Color Key and Histogram
Count
0
25
0
3e+05
Value
Heatmap of normalized counts [Euclidean dist, Ward link]
MatureLeaf_NoSi_I
MatureLeaf_NoSi_I
MatureLeaf_Si_R2
MatureLeaf_Si_R3
MatureLeaf_NoSi_I
MatureLeaf_Si_R1
Root_NoSi_R3
Root_Si_R3
Root_NoSi_R2
Root_Si_R1
Root_Si_R2
Root_NoSi_R1
Root_NoSi_R1
Root_Si_R2
Root_Si_R1
Root_NoSi_R2
Root_Si_R3
Root_NoSi_R3
MatureLeaf_Si_R1
atureLeaf_NoSi_R2
MatureLeaf_Si_R3
MatureLeaf_Si_R2
atureLeaf_NoSi_R1
atureLeaf_NoSi_R3
D
PCA on normalized counts
100
0
-100
-200
18.43 %
-100
0
100
61.72 %
Biological_Factors
MatureLeaf_NoSi
MatureLeaf_Si
Root_NoSi
Root_Si
