# Supplementary figures and images for: DiCoExpress: a tool to process multifactorial RNAseq experiments from quality controls to co-expression analysis through differential analysis based on contrasts inside GLM models
Source: Plant Methods. 2020 May 12;16:68. doi: 10.1186/s13007-020-00611-7 (PMC7216733; doi:10.1186/s13007-020-00611-7)

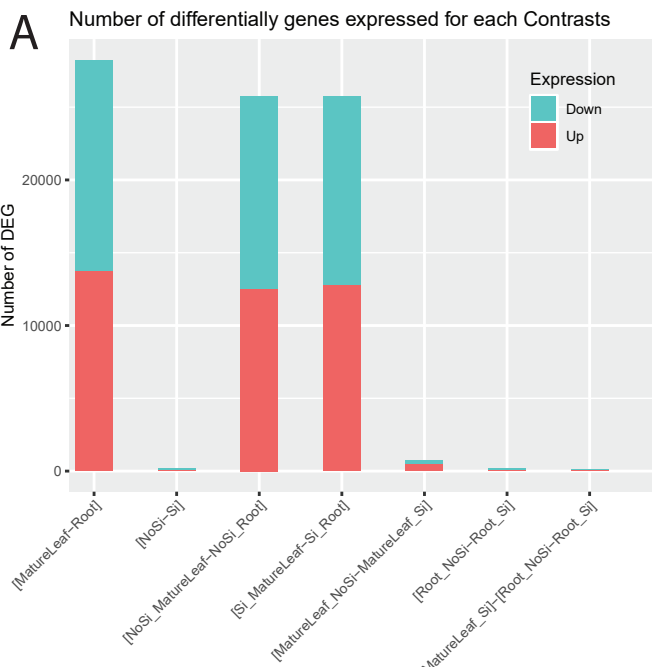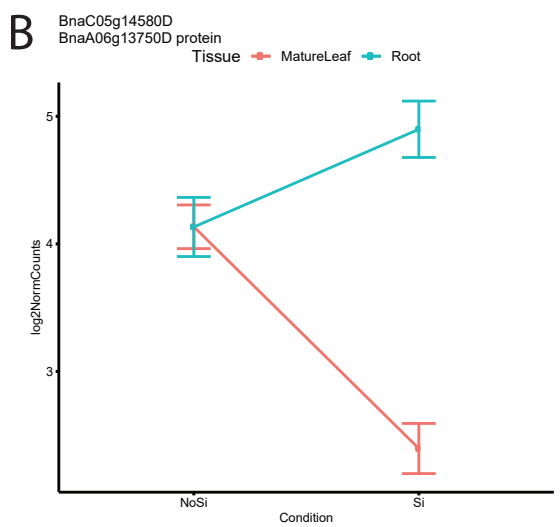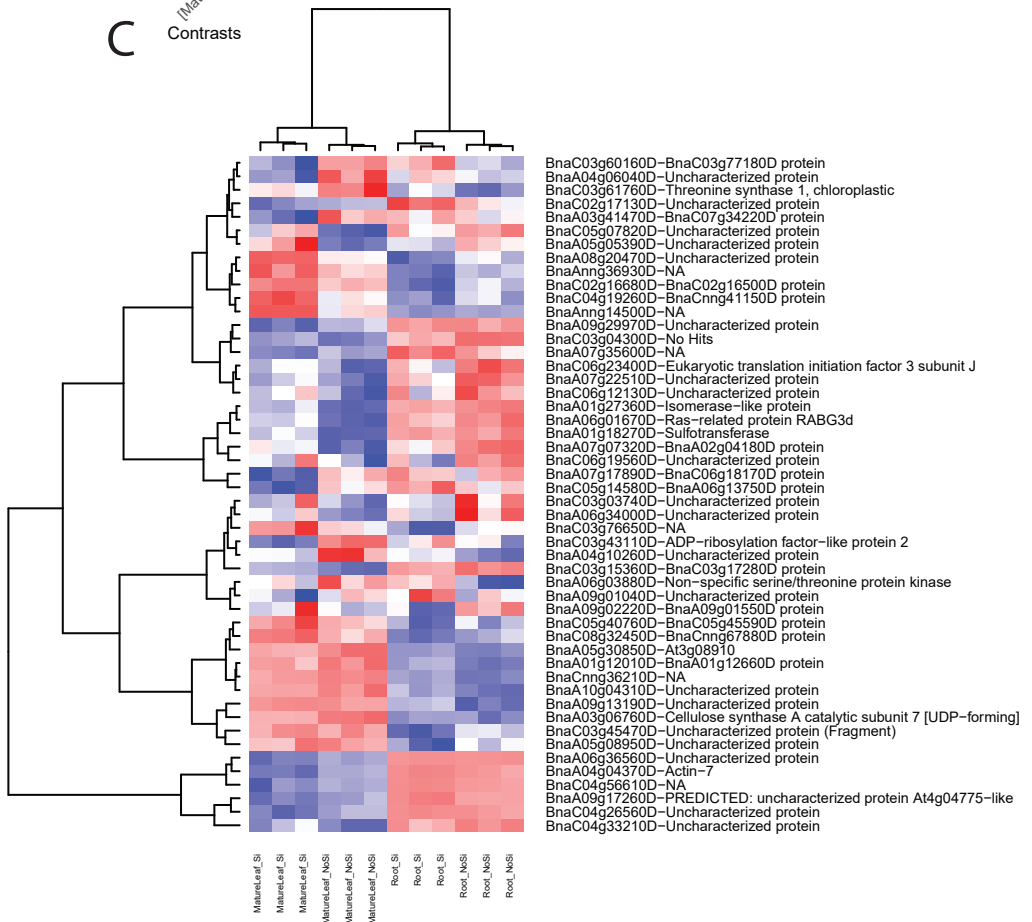

Supplement: Supplementary file 3 — Additional file 3. Differential expression Brassica napus analysis results. Differential expression analysis results with (A) the barplot of Up and Down differentially expressed genes for each contrast, (B) an example of one gene differentially expressed in the interaction contrast. (C) Hierarchical clustering of the top 50 DEGs for the interaction contrast. [file 13007_2020_611_MOESM3_ESM.pdf]
