# Supplementary material for: DiCoExpress: a tool to process multifactorial RNAseq experiments from quality controls to co-expression analysis through differential analysis based on contrasts inside GLM models
Source: Plant Methods. 2020 May 12;16:68. doi: 10.1186/s13007-020-00611-7 (PMC7216733; doi:10.1186/s13007-020-00611-7)
Supplement: Supplementary file 4 — Additional file 4. Analysis Tutorial. [file 13007_2020_611_MOESM4_ESM.pdf]

DiCoExpress: how to use the functions

ANALYSIS TUTORIAL USING THE BRASSICA NAPUS RNASEQ DATASET

Ilana Lambert, Christine Paysant-Le-Roux, Stefano Colella and Marie-Laure Martin-Magniette

2020-04-09

Aim of this document

This tutorial shows how to run an RNAseq analysis with DiCoExpress by using a RNAseq transcriptome dataset published by Haddad et al. (2019). In this study, the authors studied the effects of silicon supply on the root and mature leaf transcriptome in Brassica napus L.

Once the user added the required file in the Data directory and modified the parameter `Project_Name`, the script can be used without any parameter modification until the enrichment analysis of the DEG lists. Then the user has to specify some contrasts if he want to perform a coexpression analysis.

Advanced users can also customize some parts of the script for their analyses by modifying some parameters. These arguments are found before the command line to call a given function. Please, refer to the Reference Manual for details on argument options.

Dataset description

This dataset contains root and mature leaf expression data from B. napus with or without silicon treatment (Si). Three biological replicates are available for each data point. The two input files are available in the directory DiCoExpress/Data. We also added the annotation of B. napus v.5 downloaded from the Brassica Genome database in this same directory as well as a GOSLIM file to perform enrichment tests on groups of genes.

To begin

The Template_scripts directory has to be the R working directory. The script associated with an analysis must be placed in this Template_scripts directory.

To load the functions and all the R-packages required by the DiCoExpress and to specify the paths of the Data and Results directories, the user has to run these lines of code:

```
source("../Sources/Load_Functions.R")
Load_Functions()

Working_Directory <- ".."
Data_Directory <- paste0(Working_Directory,"/Data")
Results_Directory <- paste0(Working_Directory,"/Results")
```

Load input files

The `Project Name` allows DiCoExpress to find and load all input files of the given project. A filter option is available to choose a subset of samples. By default, `filter = NULL` and the whole dataset is analyzed.

```
Project_Name <- "Brassica_napus"
Filter=NULL
Sep=","

Data_Files <- Load_Data_Files(Data_Directory, Project_Name,
                    Filter, Sep)

Project_Name <- Data_Files$Project_Name
Target <- Data_Files$Target
Raw_Counts <- Data_Files$Raw_Counts
Annotation <- Data_Files$Annotation
Reference_Enrichment<-Data_Files$Reference_Enrichment
```

This function returns a list of 4 elements that are then stored in four different R objects. They correspond to the Project_Name, the Target table, the Raw Counts table, and, if available, the Annotation table and the Reference_Enrichment table.

The annotation file gives an annotation for each gene, added in the output files. The Reference_Enrichment file is required to perform enrichment analyses. See the Reference Manual for a detailed description of these two files.

[Optional] Using the filter argument: an example

The filter argument is a list of filter rules and a new project name. Each filter rule is described by 3 characters: the name of the factor, the level of this factor and TRUE or FALSE. TRUE means that only the level of this factor is kept, and FALSE means that it is removed. The last element of the list is a string of characters to give a name to the project on the filtered dataset.

```
Filter=list(c("Name_of_factor", "level_of_this_factor", TRUE/FALSE),
                "New_Project_Name")
```

In the B. napus dataset, to focus only on the root samples, without modifying the input files, the user can use `Filter=list(c("Tissue", "Root", TRUE),"Brassica_napus_Root")`

Be careful: when a factor has more than two modalities, TRUE means that only the mentioned level is kept and FALSE means that the mentioned level is removed.

Quality control

This step is to assess data quality. Low expressed genes are filtered such that only genes whose cpm expression is greater than `CPM_Cutoff` in x samples are kept. The value x is given by `Filter_Strategy`. The method for the correction of the library sizes is given by `Normalization_Method`. DiCoExpress gives a color per conditions, but the user can choose the colors used for the sample groups with `Color_Group`.

For the B. napus dataset, we used

```
Filter_Strategy="NbConditions"
CPM_Cutoff=1
Normalization_Method="TMM"
Color_Group=c("darkolivegreen3","darkgreen","tan3","darkorange4")

Quality_Control(Data_Directory, Results_Directory, Project_Name,
```

```
            Target, Raw_Counts, Filter_Strategy,
            Color_Group, CPM_Cutoff,
            Normalization_Method)
```

The QualityControl function returns 3 output files saved in the directory Results/Brassica_napus/QualityControl:

- **Brassica_napus_Normalization_Results.txt**
- **Brassica_napus_Low_count_genes.txt**
- **Brassica_napus_Data_Quality_Control.pdf**

The number of genes discarded by the filtering step is available in the output file **Brassica_napus_Normalization_Results.txt** or can be directly visualized in the console pane:

```
## ################################################
## Filtering
## ################################################
##
## #### Description of Raw counts table ####
## Number of samples: 12
## Number of genes: 52962
##
## Number of genes discarded by the filtering: 9233
## Number of genes analyzed after filtering: 43729
##
## ################################################
##  Statistics on the normalization factors
## ################################################
##
##    Min. 1st Qu.  Median    Mean 3rd Qu.    Max.
##  0.7367  0.7878  1.0351  1.0321  1.2851  1.3152
```

```
## png
##   2
```

Differential expression analysis

The generalized linear model used to perform differential expression analysis is defined with the GLM_Contrasts function by specifying the arguments `Replicate` and `Interaction`.

Let μ_{tcr} be the log of the mean expression of a gene in Tissue t treated with Treatment c for the Replicate r.

- If `Replicate=FALSE` and `Interaction=FALSE`,

$$\mu_{tcr} = Intercept + T_t + C_c$$

- If `Replicate=TRUE` and `Interaction=FALSE`,

$$\mu_{tcr} = Intercept + T_t + C_c + R_r$$

- If `Replicate=FALSE` and `Interaction=TRUE`,

$$\mu_{tcr} = Intercept + T_t + C_c + (TC)_{tc}$$

- If `Replicate=TRUE` and `Interaction=TRUE`,

$$\mu_{tcr} = Intercept + T_t + C_c + R_r + (TC)_{tc}$$

where T_t states for the effect of Tissue, C_c the treatment effect, R_r the replicate effect and $(TC)_{tc}$ the interaction

between Tissue and Treatment.

When two biological factors are available, an interaction term can be added. The interaction analysis often allows answering more directly to a biological question, and the user avoids a long interpretation work by comparing different lists of differentially expressed genes.

For the B. napus dataset, we used the glm with `Replicate=TRUE` and `Interaction=TRUE`.

```
Replicate=TRUE
Interaction=TRUE

Model <- GLM_Contrasts(Results_Directory, Project_Name,
                       Target, Replicate, Interaction)

GLM_Model <- Model$GLM_Model
Contrasts <- Model$Contrasts
```

The model and the contrasts are available in the directory Results/Brassica_napus/DiffAnalysis:

- **Brassica_napus_GLM_Model.txt**
- **Brassica_napus_Contrasts_Matrix.txt**
- **Brassica_napus_GLM_Contrasts.txt**: useful to choose the contrasts of interest

By default, the differential analysis is performed on all the contrasts automatically generated. We point out that some of them might not be biologically relevant to interpret. To only calculate a subset of contrasts, the user can specify them by using the `Index_Contrast` parameter. The number associated with each contrast is available in the output file **Brassica_napus_GLM_Contrasts.txt**.

```
Index_Contrast=1:nrow(Contrasts)
Alpha=0.05
NbGenes_Profiles=20
NbGenes_Clustering=50

DiffAnalysis.edgeR(Data_Directory, Results_Directory, Project_Name,
            Target, Raw_Counts, GLM_Model, Contrasts,
            Index_Contrast, Filter_Strategy, Alpha,
            NbGenes_Profiles, NbGenes_Clustering,
            CPM_Cutoff, Normalization_Method)
```

The DiffAnalysis_edgeR function returns 9 output files saved in the directory Results/Brassica_napus/DiffAnalysis:

- **Brassica_napus_Compare_table.txt**
- **Brassica_napus_Contrasts_Interests_Matrix.txt**
- **Brassica_napus_DiffAnalysis_Comparisons.txt**
- **Brassica_napus_Down_Up_DEG.pdf**
- **Brassica_napus_Raw_pvalues_histograms.pdf**
- **Brassica_napus_Estimated_Dispersion.txt**
- **Brassica_napus_Fitted_Values.txt**
- **Brassica_napus_NormCounts_log2.txt**
- **Brassica_napus_NormCounts_log2_Mean_SD.txt**

The number of differentially expressed genes for each contrast can be found in the output file **Brassica_napus_DiffAnalysis_Comparisons.txt** or directly visualized in the console pane.

It is important to look at the raw p-values histograms available in Project_Name_Raw_pvalues_histograms.pdf to check the quality of the differential analysis for each contrast of interest.

For illustration, we look at the histograms of raw p-values for [MatureLeaf-Root] and [NoSi-Si] contrasts available in **Brassica_napus_Raw_pvalues_histograms.pdf**:

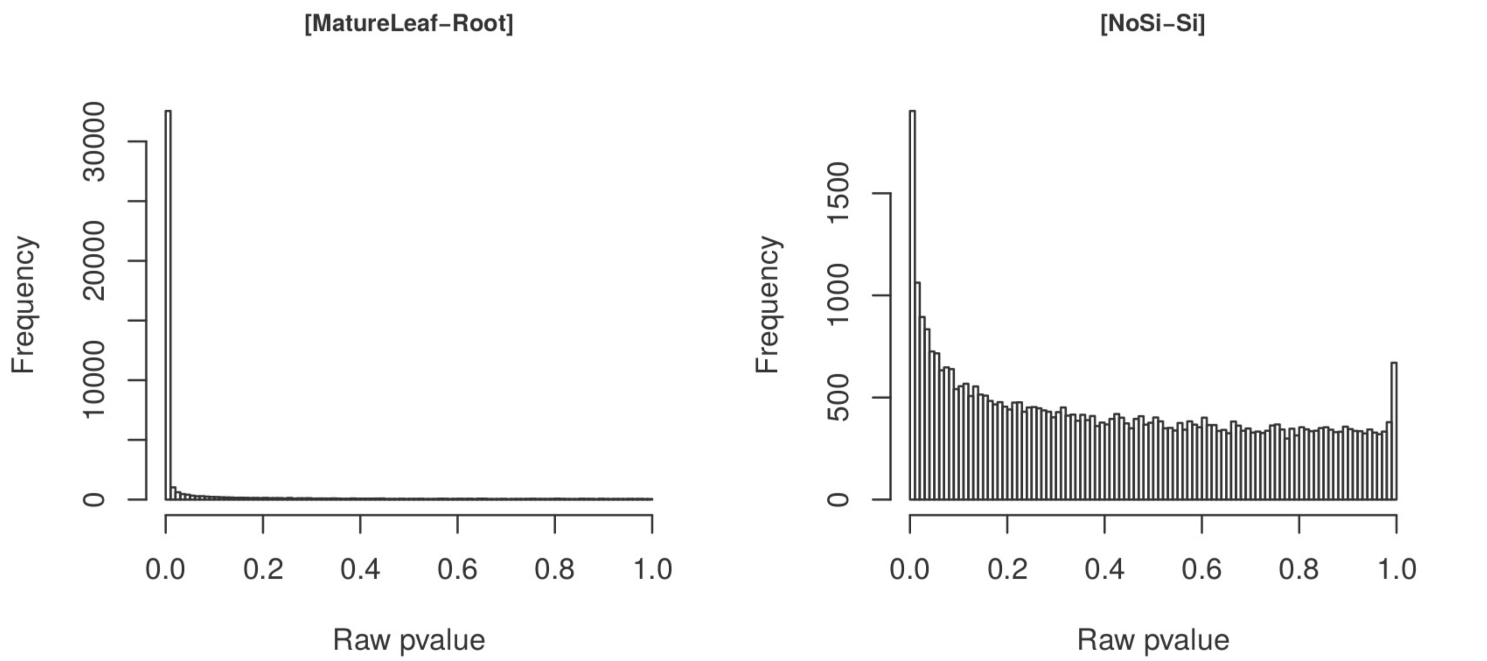

The histogram of raw p-values for the [MatureLeaf-Root] contrast is as expected whereas we observe a peak for the [NoSi-Si] contrast around 1. As a consequence, the results are not reliable.

One solution to correct this distribution is to choose a more stringent CPM cutoff. In our example, we re-run the analysis using `CPM_Cutoff = 5` instead of `CPM_Cutoff = 1`, getting reliable results for differential analysis of the B. napus dataset.

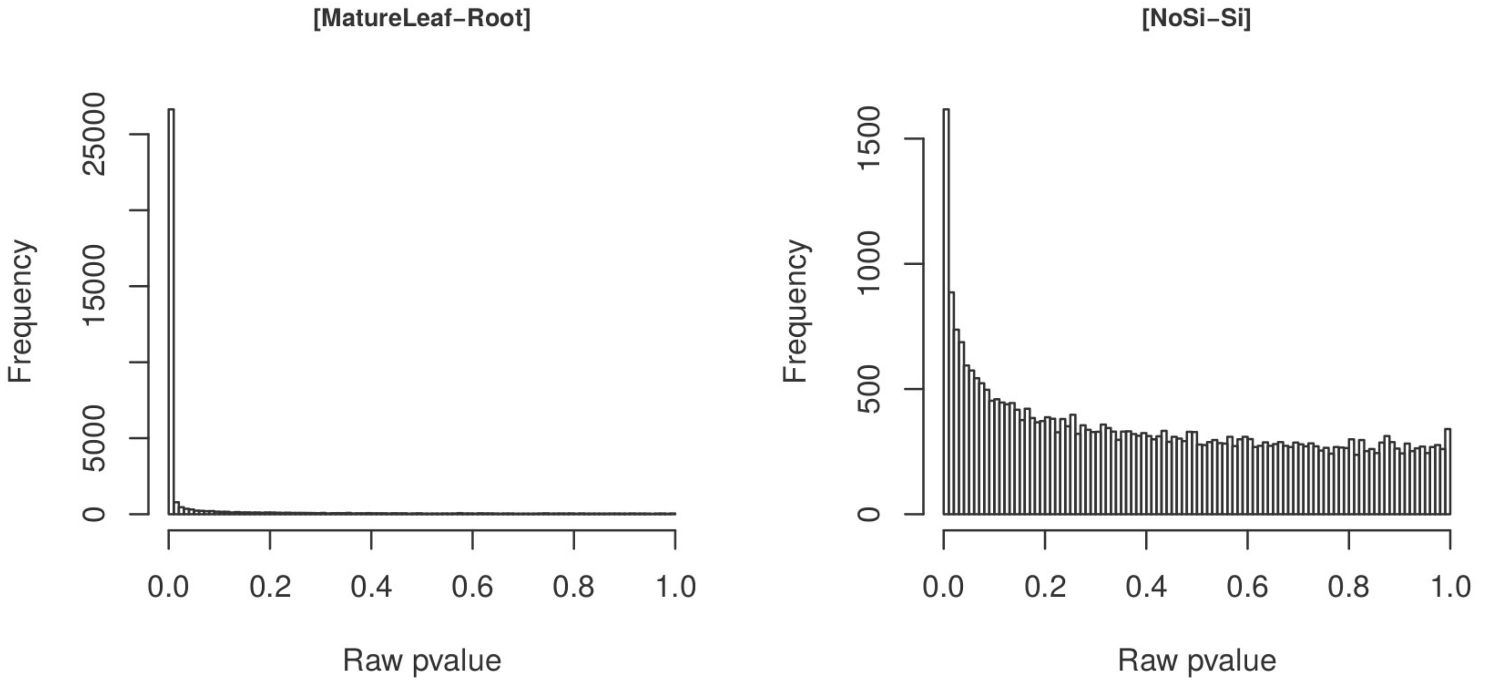

If the distribution of the raw p-values remains unsatisfactory, the problem might come from the fact that the number of parameters is too large compared to the number of observations available to estimate them. In this case, we advise removing the interaction term in the generalized linear model.

For each contrast of interest, a subdirectory is created. For the B. napus dataset, they are named:

- **[MatureLeaf-Root]**
- **[NoSi-Si]**
- **[NoSi_MatureLeaf-NoSi_Root]**
- **[Si_MatureLeaf-Si_Root]**
- **[MatureLeaf_NoSi-MatureLeaf_Si]**
- **[Root_NoSi-Root_Si]**
- **[MatureLeaf_NoSi-MatureLeaf_Si]-[Root_NoSi-Root_Si]**

And each one contains 6 files. For illustration, for the contrast [MatureLeaf-Root], they are named:

- **Brassica_napus_[MatureLeaf-Root]_LRT_BH.txt**
- **Brassica_napus_[MatureLeaf-Root]_DEG_BH.txt**
- **Brassica_napus_[MatureLeaf-Root]_id_DEG.txt**
- **Brassica_napus_[MatureLeaf-Root]_plotSmear.pdf**
- **Brassica_napus_[MatureLeaf-Root]_Top20_Profile.pdf**
- **Brassica_napus_[MatureLeaf-Root]_Top50_Clustering.pdf**

Enrichment on DEG lists

If a reference enrichment file (GO terms in this example) has been saved in the Data directory, the enrichment analysis can be performed on all the list of DEGs as follows:

```
Title=NULL
Alpha_Enrichment=0.01

Enrichment(Results_Directory, Project_Name, Title,
                  Reference_Enrichment, Alpha_Enrichment)
```

The enrichment results are saved in each subdirectory of Results/Brassica_napus/DiffAnalysis/. For example, for the contrast [MatureLeaf-Root], two new files are generated:

- **Brassica_napus_[MatureLeaf-Root]_All_Enrichment_Results.txt**
- **Brassica_napus_[MatureLeaf-Root]_Significant_Enrichments.txt**

An annotation term (GO in this case) is declared enriched if its raw p-value is lower than the `Alpha_Enrichment` set value of 0.01. If the user wants to adjust the raw p-values a posteriori, they are available in the file with suffix All_Enrichment_Results.txt.

In Results/Brassica_napus/DiffAnalysis/, the file **Brassica_napus_NoSi-Si_Summary_Enrichment.txt** provides a summary of the enrichments tests useful to compare the significant annotations found for all the DEG lists.

Venn diagram and merge of DEG lists

The user could be interested in looking at the genes differentially expressed in several contrasts. We cannot automate all possible combinations, but the Venn_Intersection_Union function offers some options to combine several lists of differentially expressed genes.

The user has to

- choose a `Title` so that DiCoExpress creates a subdirectory in Results/Project_Name/Venn_Intersection_Union/.
- indicate the considered contrasts in the `Groups` argument. The list of the contrasts is available with `print(Contrats[,1])`
- specify the `Operation`
  – When `Operation="Union"`, the list of all genes differentially expressed in at least one of the contrasts considered is generated.
  – When `Operation="Intersection"`, the list of genes differentially expressed in all the contrasts considered is generated.

For the B. napus dataset, to investigate the treatment effect, we create the union list from the 3 contrasts: [MatureLeaf_NoSi-MatureLeaf_Si], [Root_NoSi-Root_Si], [MatureLeaf_NoSi-MatureLeaf_Si]-[Root_NoSi-Root_Si] and we set the `Title=NoSi-Si`.

```
Title="NoSi-Si"
Groups=c("[MatureLeaf_NoSi-MatureLeaf_Si]","[Root_NoSi-Root_Si]",
        "[MatureLeaf_NoSi-MatureLeaf_Si]-[Root_NoSi-Root_Si]")
Operation="Union"

Venn_IntersectUnion(Data_Directory, Results_Directory, Project_Name,
                    Title, Groups, Operation)
```

In this example, the Venn_IntersectUnion function returns 3 output files in the directory Results/Brassica_napus/Venn_Intersection_Union/NoSi-Si:

- **Brassica_napus_NoSi-Si_Union_List.txt**
- **Brassica_napus_NoSi-Si_Union_Summary_Table.txt**
- **Brassica_napus_NoSi-Si_Venn_Diagram.pdf**

In **Brassica_napus_NoSi-Si_Union_Summary_Table.txt** file, a column indicates the contrasts for which each gene is differentially expressed. As the contrast names are quite long, DiCoExpress uses a letter code to identify them. The legend of the groups is available in the output file **Brassica_napus_NoSi-Si_Venn_Diagram.pdf**.

Coexpression analysis

On any lists generated by the Venn_Intersection_Union function, the user can perform a co-expression analysis. The default values of `A`, `B`, and `K` are adapted for most analyses.

```
A=5
B=40
K=c(2,seq(5,30,by=5))

Coexpression_coseq(Data_Directory, Results_Directory, Project_Name,
           Title, Target, Raw_Counts, Color_Group, A, B, K)
```

The Coexpression_coseq function that we run on **Brassica_napus_NoSi-Si_Union_List.txt**, returns on the 17 output files placed in the folder Results/Brassica_napus/Coexpression/NoSi-Si

The following files are about the analysis process:

- **Brassica_napus_NoSi-Si_Loop_1.pdf**
- **Brassica_napus_NoSi-Si_Loop_2.pdf**
- **Brassica_napus_NoSi-Si_coseq_final.RData**

- **Brassica_napus_NoSi-Si_coseq_loop_2.RData**
- **Brassica_napus_NoSi-Si_Results_First_Loop.txt**
- **Brassica_napus_NoSi-Si_Results_Second_Loop.txt**

If the minimum value of the ICL curve is clearly identifiable, then the quality of the coexpression analysis is good. For B. napus dataset, we observe a clear minimum at 7 in the **Brassica_napus_NoSi-Si_Loop_2.pdf**:

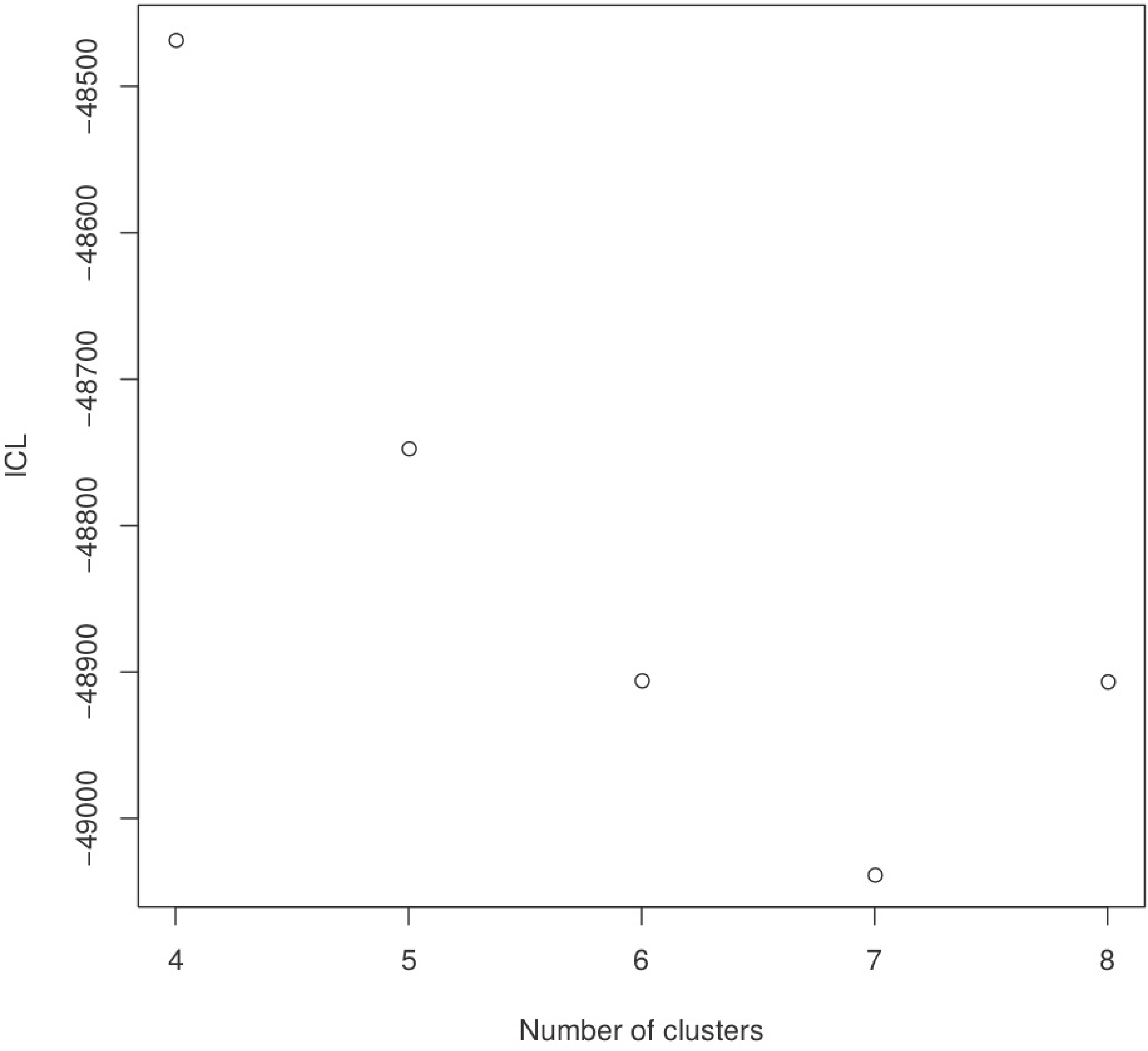

The coexpression analysis generates several files containing the results:

- **Brassica_napus_NoSi-Si_AllClusters.txt**
- **Brassica_napus_NoSi-Si_Final_Coseq.pdf**
- **Brassica_napus_NoSi-Si_Results_Final.txt**
- **Brassica_napus_NoSi-Si_Boxplot_profiles_Coseq.pdf**
- **Brassica_napus_NoSi-Si_Cluster1_GeneID.txt**
- **Brassica_napus_NoSi-Si_Cluster2_GeneID.txt**
- **Brassica_napus_NoSi-Si_Cluster3_GeneID.txt**
- **Brassica_napus_NoSi-Si_Cluster4_GeneID.txt**
- **Brassica_napus_NoSi-Si_Cluster5_GeneID.txt**

- **Brassica_napus_NoSi-Si_Cluster6_GeneID.txt**
- **Brassica_napus_NoSi-Si_Cluster7_GeneID.txt**

Enrichment of the coexpression clusters

Enrichment tests are performed on all the co-expressed groups found in the coexpression analysis using

```
Enrichment(Results_Directory, Project_Name, Title,
                Reference_Enrichment, Alpha_Enrichment)
```

For our example, the results are saved in Results/Brassica_napus/Coexpression/NoSi-Si. For each cluster, there are 2 files, one with all the results and a second with only the significant enrichments (raw p-value < Alpha_Enrichment)

- **Brassica_napus_NoSi-Si_Cluster_1_All_Enrichment_Results.txt**
- **Brassica_napus_NoSi-Si_Cluster_1_Significant_Enrichments.txt**
- **Brassica_napus_NoSi-Si_Cluster_2_All_Enrichment_Results.txt**
- **Brassica_napus_NoSi-Si_Cluster_2_Significant_Enrichments.txt**
- **Brassica_napus_NoSi-Si_Cluster_3_All_Enrichment_Results.txt**
- **Brassica_napus_NoSi-Si_Cluster_3_Significant_Enrichments.txt**
- **Brassica_napus_NoSi-Si_Cluster_4_All_Enrichment_Results.txt**
- **Brassica_napus_NoSi-Si_Cluster_4_Significant_Enrichments.txt**
- **Brassica_napus_NoSi-Si_Cluster_5_All_Enrichment_Results.txt**
- **Brassica_napus_NoSi-Si_Cluster_5_Significant_Enrichments.txt**
- **Brassica_napus_NoSi-Si_Cluster_6_All_Enrichment_Results.txt**
- **Brassica_napus_NoSi-Si_Cluster_6_Significant_Enrichments.txt**
- **Brassica_napus_NoSi-Si_Cluster_7_All_Enrichment_Results.txt**
- **Brassica_napus_NoSi-Si_Cluster_7_Significant_Enrichments.txt**

DiCoExpress performs an enrichment test performed also on all the classified genes whatever their cluster membership:

- **Brassica_napus_NoSi-Si_AllClusters_All_Enrichment_Results.txt**
- **Brassica_napus_NoSi-Si_AllClusters_Significant_Enrichments.txt**

Finally, a summary the of the significant enrichments found across all the clusters is presented in the file:

- **Brassica_napus_NoSi-Si_Summary_Cluster_Enrichment.txt**

Parameter and Session information

The **SessionInfo.txt** file in Results/Brassica_napus gets information about the versions of R, the OS and all the required packages, including the version used for the analysis.

The **Parameter_Information.txt** file recalls the values of the critical parameters used in the script for the given analysis.
