# Supplementary material for: DiCoExpress: a tool to process multifactorial RNAseq experiments from quality controls to co-expression analysis through differential analysis based on contrasts inside GLM models
Source: Plant Methods. 2020 May 12;16:68. doi: 10.1186/s13007-020-00611-7 (PMC7216733; doi:10.1186/s13007-020-00611-7)
Supplement: Supplementary file 5 — Additional file 5.Brassica napus GLM contrasts results. Organisation of the genes impacted in their transcription by the silicon treatment issued from the GLM differential analysis. (A) Venn diagram describing the numbers of differentially expressed genes in the three contrasts [MatureLeaf_NoSi-MatureLeaf_Si], [Root_NoSi-Root_Si] and [MatureLeaf_NoSi-MatureLeaf_Si]-[Root_NoSi-Root_Si], and their overlaps: (B) Gene expression of genes representative of each group of the Venn diagram (described with small letters from a to g). [file 13007_2020_611_MOESM5_ESM.pdf]

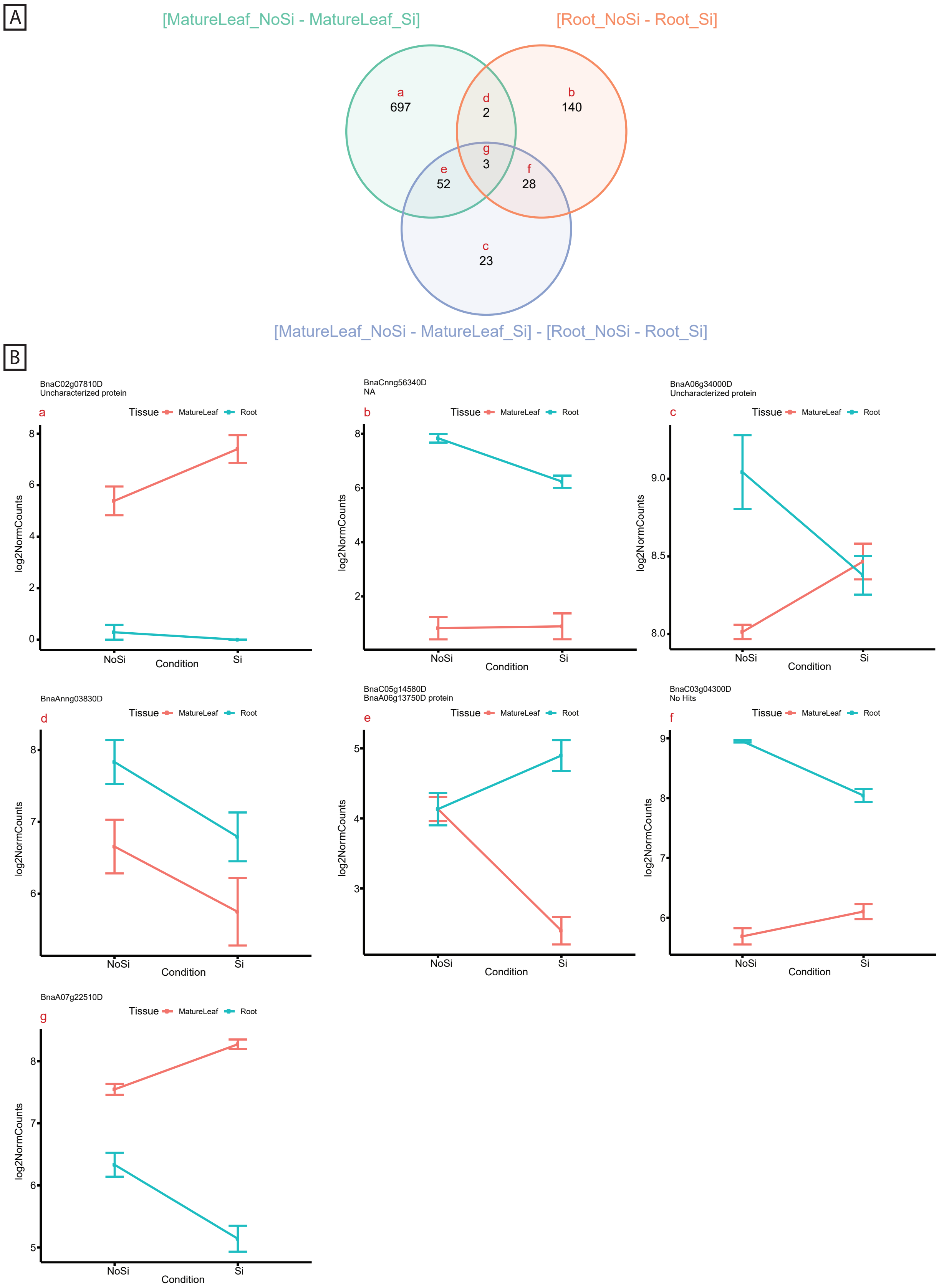
A
[MatureLeaf_NoSi - MatureLeaf_Si]
[Root_NoSi - Root_Si]
a 697
d 2
b 140
g 3
e 52
f 28
c 23
[MatureLeaf_NoSi - MatureLeaf_Si] - [Root_NoSi - Root_Si]
B
BnaC02g07810D
Uncharacterized protein
BnaCnng56340D
NA
BnaA06g34000D
Uncharacterized protein
BnaAnng03830D
BnaC05g14580D
BnaA06g13750D protein
BnaC03g04300D
No Hits
BnaA07g22510D
Tissue MatureLeaf Root
log2NormCounts
Condition
NoSi
Si
