# Supplementary material for: DiCoExpress: a tool to process multifactorial RNAseq experiments from quality controls to co-expression analysis through differential analysis based on contrasts inside GLM models
Source: Plant Methods. 2020 May 12;16:68. doi: 10.1186/s13007-020-00611-7 (PMC7216733; doi:10.1186/s13007-020-00611-7)
Supplement: Supplementary file 6 — Additional file 6. Comparison of DEG lists and clusters of co-expression. [file 13007_2020_611_MOESM6_ESM.pdf]

		Groups of differentially expressed genes (Venn diagram)							
**Clusters of co-expression**		**a**	**b**	**c**	**d**	**e**	**f**	**g**	**Total**
	**0**	1	2	0	0	0	0	0	**3**
	**1**	194	0	0	0	9	0	0	**203**
	**2**	41	25	8	1	4	5	1	**85**
	**3**	47	12	2	1	1	8	0	**71**
	**4**	112	15	4	0	10	4	1	**146**
	**5**	100	13	4	0	10	3	0	**130**
	**6**	105	1	0	0	0	0	0	**106**
	**7**	97	72	5	0	18	8	1	**201**
	**Total**	**697**	**140**	**23**	**2**	**52**	**28**	**3**	**945**
